# Meta-analysis of intrauterine hCG perfusion efficacy in recurrent implantation failure as defined by ESHRE guidelines

**DOI:** 10.1186/s12884-024-06662-1

**Published:** 2024-07-09

**Authors:** Xi Luo, Yuerong Wu, Yongfang Xu, Lujuan Rong, Xiaoping Liu, Xiaoting Zhou, Yun Bai, Ze Wu

**Affiliations:** 1 Department of Reproductive Medicine, NHC Key Laboratory of Healthy Birth and Birth Defect Prevention in Western China, The First People’s Hospital of Yunnan Province, Kunming, China; 2 Reproductive Medical Center of Yunnan Province, The Affiliated Hospital of Kunming University of Science and Technology, Kunming, China; 3 Faculty of Life science and Technology, Kunming University of Science and Technology, Kunming, China; 4 Medical school, Kunming University of Science and Technology, Kunming, China

**Keywords:** Human chorionic gonadotropin, Recurrent implantation failure, Assisted reproductive technology, Embryo implantation, Clinical pregnancy rate

## Abstract

**Purpose:**

This study evaluates the efficacy of intrauterine hCG perfusion for RIF, as defined by ESHRE 2023 guidelines, highlighting hCG as a cost-effective alternative to other immunotherapies, especially suitable for less developed regions. It aims to clarify treatment guidance amidst previous inconsistencies.

**Methods:**

This meta-analysis, registered with PROSPERO (CRD42024443241) and adhering to PRISMA guidelines, assessed the efficacy and safety of intrauterine hCG perfusion in enhancing implantation and pregnancy outcomes in RIF. Comprehensive literature searches were conducted through December 2023 in major databases including PubMed, Web of Science, Embase, the Cochrane Library, and key Chinese databases, without language restrictions. Inclusion and exclusion criteria were strictly aligned with the 2023 ESHRE recommendations, with exclusions for studies lacking robust control, clear outcomes, or adequate data integrity. The risk of bias was evaluated using the Newcastle-Ottawa Scale, ROBINS-I, and RoB2 tools. Data analysis was performed in R using the ‘meta’ package, employing both fixed and random effect models to account for study variability. Subgroup analyses by dosage, volume, hCG concentration, timing of administration, and type of embryo transfer were conducted to deepen insights, enhancing the reliability and depth of the meta-analysis in elucidating the role of hCG perfusion in RIF treatments.

**Results:**

Data from 13 studies, comprising six retrospective and six prospective studies from single centers, along with one multi-center RCT, totaling 2,157 participants, were synthesized to evaluate the effectiveness of intrauterine hCG perfusion in enhancing implantation and pregnancy outcomes in patients with RIF. Significant improvements were observed in clinical pregnancy and embryo implantation rates across various dosages, timing of administration, and embryo developmental stages, without impacting miscarriage rates. Notably, the most significant efficacy within subgroups occurred with a 500 IU dosage and perfusion parameters of ≤ 500µL volume and ≥ 2 IU/µL concentration. Additionally, a limited number of studies showed no significant increases in ectopic pregnancy or multiple pregnancy rates, and a modest improvement in live birth rates, although the small number of these studies precludes definitive conclusions.

**Conclusions:**

The analysis suggests that intrauterine hCG perfusion probably enhances embryo implantation, clinical pregnancy, and live birth rates slightly in RIF patients. Benefits are indicated with a dosage of 500 IU and a maximum volume of 500µL at concentrations of at least 2 IU/µL. However, substantial heterogeneity from varying study types and the limited number of studies necessitate cautious interpretation. These findings underscore the need for more rigorously designed RCTs to definitively assess the efficacy and safety.

**Supplementary Information:**

The online version contains supplementary material available at 10.1186/s12884-024-06662-1.

## Introduction

Recurrent Implantation Failure presents a significant challenge in the realm of ART, affecting up to 10% of individuals undergoing IVF [1]. Traditionally, RIF has been defined as the repeated transfer of good-quality embryos into a healthy uterus without achieving successful implantation and pregnancy. This definition, however, has lacked uniformity across the field, leading to variances in clinical approaches and research methodologies. It wasn’t until the ESHRE’s 2023 Recurrent Implantation Failure practice recommendations that a more standardized definition emerged [2]. ESHRE now defines RIF as the failure to achieve clinical pregnancy after at least three embryo transfers of good quality, particularly in younger patients. This pivotal development in defining RIF has profound implications for the diagnosis, treatment, and research of this condition in the field of reproductive medicine.

The complexity of RIF stems from its multifactorial nature, with contributing factors ranging from embryonic quality and endometrial receptivity to maternal immune system issues and uterine abnormalities [3–6]. Despite advancements in ART, the precise mechanisms and optimal treatments for RIF remain areas of active investigation. This ongoing quest for understanding and effective interventions brings us to the exploration of innovative treatments, among which is the role of hCG in the implantation process [7].

Garnering significant interest in the scientific community, the potential use of hCG in intrauterine perfusion treatments for RIF represents a promising avenue [8]. This approach is rooted in the hormone’s ability to modulate key factors involved in implantation, such as endometrial matrix-metalloproteinases, growth factors, and cytokines [9–11]. Importantly, compared to other immunotherapies, hCG treatment stands out for its lower cost, making it particularly appealing for broader application in economically less developed regions. However, the path to clarity is not straightforward. Studies investigating the effectiveness of intrauterine hCG injection prior to embryo transfer have produced inconsistent results [12, 13]. This inconsistency has sparked a series of meta-analyses aimed at providing a more comprehensive understanding of hCG’s impact on IVF/ICSI outcomes.

In response to these inconsistencies and the evolving understanding of RIF, we conducted a comprehensive meta-analysis that aligns with the ESHRE’s 2023 guidelines. Our study meticulously includes studies conforming to the ESHRE’s more stringent definition of RIF, ensuring a higher degree of homogeneity and enhancing the validity of our findings. Specifically, we focus on evaluating the clinical benefits of intrauterine hCG perfusion in patients with RIF as defined by the ESHRE’s criteria.

This research represents a significant step in addressing the nuances of RIF treatment, aiming to provide clearer guidance for clinicians and improve the success rates of ART procedures. By aligning our analysis with the latest ESHRE recommendations, we aim to offer a more accurate assessment of the efficacy of hCG intrauterine perfusion in treating RIF, contributing to the optimization of treatment strategies for couples facing the challenges of RIF.

## Methods

### Search strategy

This meta-analysis, adhering to the PRISMA guidelines, was meticulously planned and executed. It has been registered with PROSPERO under the registration number CRD42024443241, ensuring its methodological rigor is transparent and adheres to international standards. Our comprehensive literature search spanned several major databases, including PubMed, MEDLINE, Embase, the Cochrane Library, and Web of Science, as well as key Chinese databases such as CNKI, Wanfang, and Weipu Database, concluding in December 2023. This approach ensured a broad and inclusive capture of relevant studies for analysis.

A detailed and extensive search strategy was implemented, using a combination of MeSH and a wide range of free-text term variants. The primary terms “human chorionic gonadotropin”, “recurrent implantation failure” and “intrauterine perfusion” were employed alongside numerous variants to ensure the capture of all relevant literature. This approach was instrumental in identifying a comprehensive array of studies encompassing various aspects of the research topic.

Additional search terms were incorporated to cover areas related to fertility treatments and outcomes, including “embryo transfer”, “fertility”, “infertility”, “assisted reproductive technology”, “pregnancy”, “miscarriage”, “implantation”, “intracytoplasmic sperm injection” and “in vitro fertilization”.

The search strategy was not confined by language or study design, aiming to include both retrospective and prospective studies as well as RCTs. This inclusive and broad approach was designed to gather a complete picture of the research landscape regarding the efficacy of intrauterine human chorionic gonadotropin perfusion in recurrent implantation failure. The manual review of reference lists from identified articles further expanded the search, ensuring no significant study was overlooked.

### Inclusion and exclusion criteria

A stringent set of inclusion and exclusion criteria was established, aligning with the 2023 ESHRE recommendations. The study focused on cases where RIF was defined as at least three failed implantations with high-quality embryos.

Exclusion criteria were carefully defined to ensure the scientific rigor of the studies included in this review. We excluded studies lacking a proper control group, such as those without a placebo or an appropriate comparator. Additionally, studies were omitted if they did not report clear primary outcomes or failed to demonstrate sufficient data integrity. For RCTs and prospective cohort studies, we specifically excluded those with inadequate randomization and blinding. In contrast, for retrospective studies, we focused on the appropriateness of study design and execution given their inherent methodological limitations. This selective approach ensured that only high-quality research was included across all study designs. To maintain publication quality, unpublished manuscripts and non-peer-reviewed articles were excluded. Furthermore, we eliminated studies with overlapping datasets to avoid redundancy and ensure the uniqueness of each study’s contribution to the meta-analysis.

### Data extraction

Data extraction was meticulously conducted to ensure accuracy and reliability in the meta-analysis. This process was carried out independently by two researchers, providing a dual-layer of scrutiny to each data point extracted from the included studies. Following their independent extractions, the data was cross-verified by a third party, Luo Xi, to further ensure precision and consistency.

In instances where discrepancies arose between the two primary extractors, the conflicts were resolved through a collaborative discussion involving all three individuals. This approach not only ensured consensus but also maintained the integrity of the data extraction process.

To assess the risk of methodology bias within the retrospective studies, the Newcastle-Ottawa Scale was employed [14]. This scale provided a systematic method to evaluate the quality of non-randomized studies, particularly in terms of selection, comparability, and exposure or outcome assessment. Utilizing the NOS scale contributed to a comprehensive and nuanced understanding of the potential biases in the studies included in this meta-analysis. Additionally, we employed the GRADE approach to assess the risk of publication bias, ensuring a robust evaluation of the evidence’s overall quality. For non-randomized control studies, the ROBINS-I tool was used to assess the risk of bias, providing further depth to our methodological scrutiny [15]. For RCTs and prospective studies, the RoB2 tool was applied, enabling a detailed and structured assessment of biases related to the randomization process, deviations from intended interventions, missing outcome data, measurement of the outcome, and selection of the reported result [16]. These combined tools ensured a thorough assessment of potential biases across different types of studies included in our meta-analysis.

Furthermore, traffic light plots were used to visually represent the bias assessments conducted using the ROBINS-I and RoB2 tools. To evaluate publication bias, funnel plots were employed to visually analyze the implantation rates, clinical pregnancy rates, and miscarriage rates, and the Egger test was conducted to examine the data.

### Primary outcomes

In assessing the efficacy of intrauterine hCG perfusion for recurrent implantation failure, a comprehensive set of outcomes was meticulously calculated to evaluate both immediate and sustained effects of the treatment. The implantation rate was derived by dividing the number of ultrasound-confirmed gestational sacs by the total number of embryos transferred. Similarly, the clinical pregnancy rate was calculated as the ratio of confirmed clinical pregnancies to the total embryo transfer cycles. The miscarriage rate, indicating early pregnancy losses of gestation, was computed as a proportion of the clinical pregnancies.

Secondary outcomes, reflecting broader reproductive outcomes and treatment implications, included the multiple pregnancy rate and ectopic pregnancy rate, both of which were calculated relative to the number of clinical pregnancies. The multiple pregnancy rate quantified the incidence of multi-fetal gestations, whereas the ectopic pregnancy rate measured pregnancies occurring outside the uterine cavity. Further, the ongoing pregnancy rate and live birth rate were assessed relative to the embryo transfer cycles, capturing the progression of pregnancies beyond the early stages and the culmination in live births, respectively.

These metrics collectively enable a nuanced analysis of the treatment’s success, addressing both immediate implantation outcomes and longer-term reproductive health impacts.

### Subgroup analysis

To enhance our understanding of the factors influencing the effectiveness of intrauterine hCG perfusion in recurrent implantation failure, we conducted a thorough subgroup analysis of various treatment protocols. This analysis meticulously assessed how differences in dosage, volume, and concentration of hCG might affect treatment outcomes. Specifically, we focused on the timing of hCG instillation, categorizing it into three key time points—three days before ET, one day before ET, and on the day of ET itself. This stratification was crucial for identifying the most effective timing for hCG administration to potentially enhance implantation rates and overall pregnancy outcomes.

In addition to timing, our analysis extended to the type of embryo transfer, contrasting fresh versus FET, given their differing physiological impacts on treatment success. We also scrutinized the variations in control group types utilized in the studies, distinguishing between blank controls, where no treatment was administered, and placebo controls, which involved the use of a substance with no therapeutic effect. This rigorous evaluation of control groups was essential to ensure the robustness of our findings and provide a clear picture of the treatment’s efficacy.

Moreover, we analyzed the developmental stages of the embryos transferred, differentiating between cleavage-stage embryos and blastocysts. This detailed subgroup analysis, however, was limited by a small number of crossover studies, which restricted our ability to explore these differences more profoundly. Nevertheless, the insights gained from these analyses have enabled us to provide targeted recommendations on the optimal protocols and characteristics that influence the success of hCG perfusion, thereby enhancing our comprehensive understanding of its efficacy in various clinical scenarios. This approach helps in tailoring treatment protocols to maximize clinical outcomes and offers a refined perspective on the nuanced variables impacting the success of treatment in cases of recurrent implantation failure.

### Statistical analysis

The statistical processing and analysis were carried out using R software, version 4.2.3. Central to this analysis was the utilization of the ‘meta’ package, a comprehensive tool within R specifically designed for conducting and facilitating meta-analyses [17]. This package was chosen for its specialized functions that are adept at handling, summarizing, and interpreting the pooled data from various studies, making it a crucial component in our analytical framework. Additionally, we employed the ‘robvis’ package for assessing the risk of bias using the ROBINS-I and RoB2 tools [18], which allowed for a structured visualization of bias across the included studies.

The analysis incorporated both the fixed effect model and the random effect model. The fixed effect model was applied under the assumption of homogeneity among study results, providing an estimate of a single shared effect size. In contrast, the random effect model was employed to account for potential variability across studies. This model is particularly valuable in meta-analyses where heterogeneity is expected in study designs, populations, or interventions. The dual application of these models enabled a robust assessment of the data, accommodating various study characteristics and ensuring a comprehensive understanding of the aggregated outcomes.

## Results

### Study characteristics

Our initial search across multiple platforms yielded 1,370 English-language publications and 84 Chinese-language publications. After an initial screening of titles and abstracts, we retained 57 articles. Following an additional evaluation of existing literature reviews, we incorporated an extra 14 articles for full-text assessment.

**Fig. 1:**
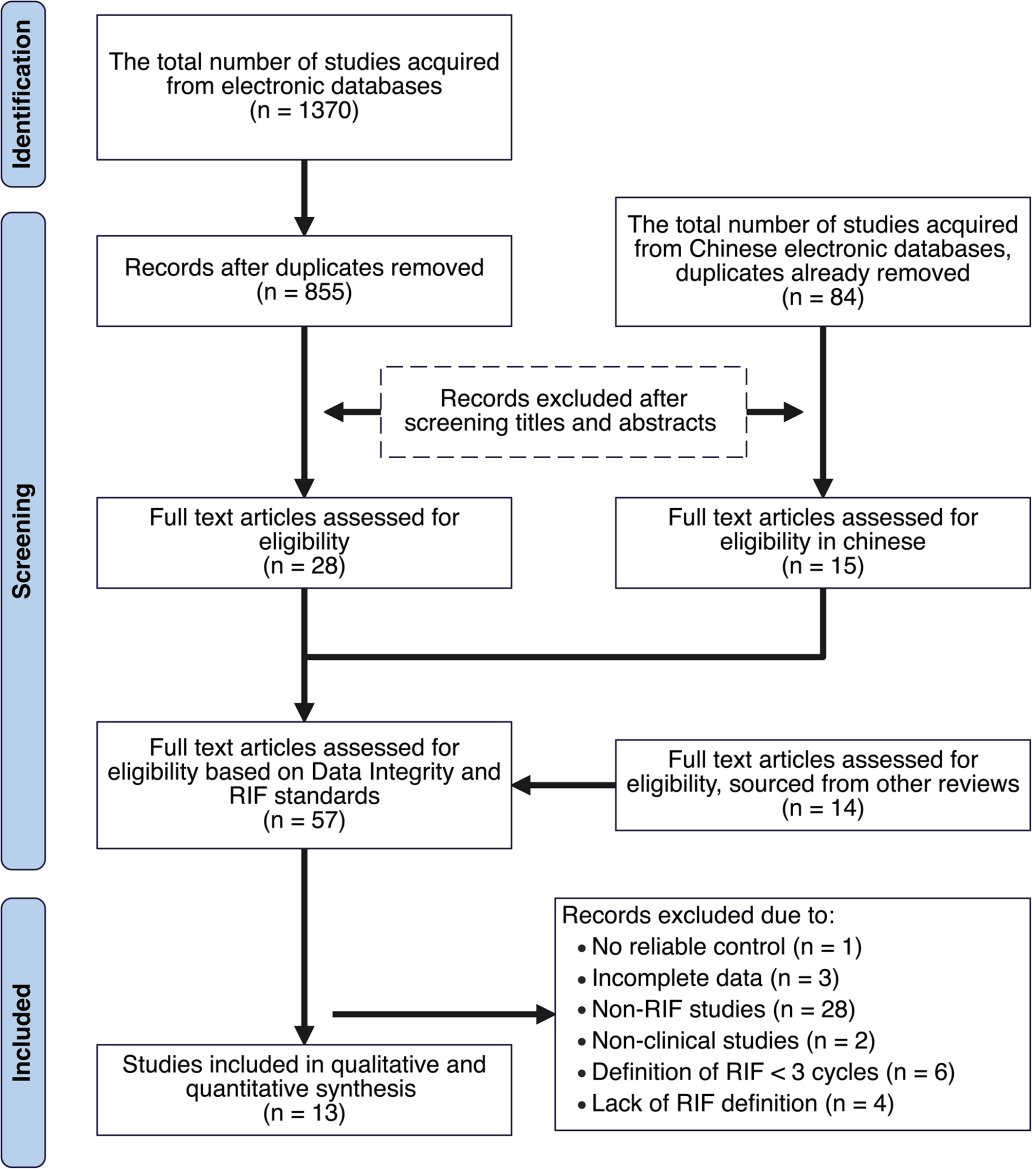
Flow chart of study selection for meta-analysis

The exclusion criteria applied to these 71 articles were as follows: lack of a reliable control (*n* = 1), incomplete data sets (*n* = 3), studies not addressing recurrent implantation failure (RIF) (*n* = 28), non-clinical studies (*n* = 2), definitions of RIF involving fewer than three cycles (*n* = 6), and studies lacking a clear definition of RIF (*n* = 4). After applying these criteria, 13 studies remained for inclusion in our meta-analysis. The selection process is visually detailed in Fig. 1, with our comprehensive search strategy outlined in the Appendix.

Of the final 13 studies included in the meta-analysis [19–31], 3 were in English and 10 in Chinese. The predominance of Chinese studies can be attributed to a significant development within the field driven by a consensus published in 2018 by Chinese societies for medical genetics and reproductive medicine, among other physician associations [32]. This consensus set forth guidelines that required at least three embryo transfer (ET) attempts with four embryos transferred each time, including at least one high-quality embryo, without achieving a clinical pregnancy. These guidelines became the standard for most intrauterine hCG perfusion trials conducted in China, ensuring that the studies adhere to contemporary clinical standards and aligning perfectly with our inclusion criteria for the meta-analysis. The high number of Chinese studies reflects the national commitment to advancing reproductive medicine practices in accordance with these expert guidelines.

The main characteristics of the studies included in our meta-analysis are methodically divided into two tables for detailed presentation. Table 1 provides a foundational overview, listing each study’s publication year, authors, participant allocation method, study type, country of study, time interval of study, definitions of RIF, and specific inclusion and exclusion criteria. This table ensures a clear understanding of the methodological and contextual framework within which each study was conducted.

**Table 1:** Characteristics of research included in the meta-analysis

Publication Year	Authors	Participant Allocation Method	Study Type	Country of Study	Time Interval of Study	RIF Definition	Inclusion Criteria	Exclusion Criteria
2015	Wen Y, et al.	Baseline Characteristics Matching	Retrospective	Guangdong, China	Apr 2012 - Sep 2014	At least 3 continuous ET attempts or 10 embryos transferred, each ET including a high-quality embryo, without clinical pregnancy.	The embryos transferred in this cycle are high-quality (Grade I and II or ≥ 3BC).	Severe uterine malformations, multiple intrauterine adhesions, chromosomal abnormalities, severe endocrine disorders or donor egg cycles.
2018	Huang PX, et al.	Baseline Characteristics Matching	Retrospective	Guangxi, China	May 2015 - July 2017	Implantation failure after 3 or more transfers of high- quality embryos.	Age ≤ 38, BMI 18–24, endometrial thickness 8–16 mm, and high-quality embryos transferred in this cycle.	Endometrial polyps, intrauterine adhesions, submucosal myomas, adenomyosis, systemic diseases, hydropic fallopian tubes, PCOS, or stage III or higher endometriosis.
2019	Wang M, et al.	Microsoft Excel ‘RAND’ Function	Prospective	Chongqing, China	Apr 2014 - Nov 2017	At least 3 ET attempts with four embryos transferred, each including a high-quality embryo, without clinical pregnancy.	Age under 40 with regular, normal menstrual periods.	Uterine abnormalities, hydrosalpinx, endometriosis, partner chromosomal abnormalities, and blastocyst-stage or genetically tested embryos.
2019	Liu XM, et al.	Computerized Random Digit Generation	Prospective	Shandong, China	Jan 2016 - Dec 2016	Implantation failure after 3 or more transfers of high- quality embryos.	Age ≤ 45, FSH < 10 IU/L, BMI 19–30 kg/m², and normal uterine cavity and normal karyotypes.	Severe uterine issues, chromosomal abnormalities, untreated hydrosalpinx, pregnancy contraindications, endocrine dysfunctions, neoplasia, significant renal or hepatic impairment, or use of interfering medications.
2020	Zhao SF, et al.	Computerized Random Digit Generation	Prospective	Not mention	Not mention	Implantation failure after at least 3 ET attempts, or four to six high-grade cleavage-stage embryos, or three or more high-grade blastocysts.	Age ≤ 38, BMI 18–24 kg/m², with two or more day-3 thawed embryos available for transfer.	Adenomyosis, endometriosis, uterine malformations, endometrial abnormalities, hydrosalpinx, or uterine adhesions.
2021	Ji XY, et al.	Computerized Random Digit Generation	Prospective	Jiangsu, China	Jan 2017 - Jun 2018	At least 3 ET attempts with four high-quality cleavage-stage embryos or two high-quality blastocyst embryos transferred, without achieving clinical pregnancy.	No history of ET difficulties, normal karyotypes, and etiology primarily due to tubal or male factors.	Moderate to severe intrauterine adhesions, hydrosalpinx, endometrial polyps, severe endocrine disorders, uterine malformations, and fibroids.
2021	Li R, et al.	Baseline Characteristics Matching	Retrospective	Guangxi, China	Jul 2017 - Jun 2019	At least 3 ET attempts with four embryos transferred, each including a high-quality embryo, without clinical pregnancy.	Age < 40, endometrial thickness > 7 mm and normal Karyotypes.	Endometrial or uterine cavity lesions, or uncontrolled endocrine or metabolic disorders.
2021	Xiong YL, et al.	Baseline Characteristics Matching	Retrospective	Guangdong, China	Jan 2018 - Dec 2019	At least 3 ET attempts with four embryos transferred, each including a high-quality embryo, without clinical pregnancy.	Age 24–40.	Acute pelvic inflammation, hydrosalpinx, vaginitis, or preoperative fever
2022	Li J, et al.	Baseline Characteristics Matching	Retrospective	Henan, China	Jan 2017 - Dec 2019	Implantation failure after 3 or more transfers.	Age ≤ 38, FSH ≤ 10 IU/L, and BMI 18–24 kg/m².	Hydrosalpinx, uterine abnormalities, chromosomal abnormalities, severe endocrine disorders, endometriosis, and donor egg cycles.
2022	Liu N, et al.	Computerized Random Digit Generation	Prospective	Hebei, China	May 2019 - May 2020	At least 3 ET attempts with four embryos transferred, each including a high-quality embryo, without clinical pregnancy.	Age < 40, normal chromosomes, normal uterine and endocrine function, transferring at least one high-quality cleavage-stage embryo this cycle.	Fallopian tube cysts, uterine fibroids, positive antiphospholipid antibodies, organic uterine lesions, hydrosalpinx, and uncontrolled endocrine or metabolic disorders.
2022	Cheng LL, et al.	Baseline Characteristics Matching	Retrospective	Hebei, China	May 2018 - Oct 2021	At least 3 ET attempts with four high-quality cleavage-stage embryos or two high-quality blastocyst embryos transferred, without achieving clinical pregnancy.	Age < 38, endometrial thickness > 7 mm and at least one high-quality cleavage-stage embryo available in this cycle.	Uterine malformations, endometriosis, adenomyosis, endometrial polyps, endometritis, hydrosalpinx, intrauterine adhesions, coagulation disorders and immune system abnormalities.
2022	Torky H, et al.	Computer Software-Based Random Allocation	RCT	Cairo, Egypt	Jan 2019 - Jan 2020	At least 3 ET attempts with four embryos transferred, without clinical pregnancy.	Age 20–39.	Medication hypersensitivity, sickle cell nephropathy, cancer history, low-quality embryos, or OHSS risk.
2023	Xu DJ, et al.	Random Digital Table Method	Prospective	Jiangxi, China	Jan 2020 - Dec 2021	At least 3 ET attempts with four embryos transferred, each including a high-quality embryo, without clinical pregnancy.	Aged 18–40.	Abnormal uterine hysteroscopy findings, chromosomal disorders, active infections, hydrosalpinx, weak ovarian reserve; systemic diseases; thyroid or thrombotic issues.

Table 2 delves into the operational specifics of the interventions and controls used in each study, including the publication year, authors, type of embryo transfer, detailed descriptions of control groups, developmental stage of embryos, timing of hCG injection, hCG volume and concentration, total number of participants in the hCG group along with their average age and number of ET attempts, and similar details for the control group. Additionally, Table 2 outlines the primary methods of endometrial preparation employed. Together, these tables provide a comprehensive summary of the key features and comparative aspects between groups, offering reproductive medicine professionals an in-depth view of the variables and conditions tested across the included studies.

**Table 2:** Study designs of research included in the meta-analysis

Publication Year	Authors	Type of Embryo Transfer	Control Group Description	Embryo Development Stage	Injection Time Point	hCG	Volume	Concentration	hCG Group Total Number	hCG Group Age	hCG Group ET Attempts	Control Group Total Number	Control Group Age	Control Group ET Attempts	Primary Endometrial Preparation
2015	Wen Y, et al.	Frozen Embryo Transfer	Blank	Cleavage & Blastocyst	ET Day	500 IU	Not mention	Not mention	104	32.5 ± 0.5	Not mention	104	32.5 ± 0.5	Not mention	NC or HRT
2018	Huang PX, et al.	Frozen Embryo Transfer	Blank	Cleavage & Blastocyst	3 + days pre-ET	1000 IU	1000 µL	1 IU/µL	77	32.66 ± 4.28	Not mention	102	Not mention	Not mention	NC or HRT
2019	Wang M, et al.	Frozen Embryo Transfer	Placebo	Cleavage	ET Day	500 IU	< 100 µL	> 5 IU/µL	69	31.35 ± 3.18	4.14 ± 0.39	68	31.7 ± 3.56	4.18 ± 0.42	HRT
2019	Liu XM, et al.	Frozen Embryo Transfer	Placebo	Cleavage & Blastocyst	3 + days pre-ET	500 IU	< 100 µL	> 5 IU/µL	152	34.83 ± 4.31	6.22 ± 1.80	151	35.25 ± 4.94	6.13 ± 1.42	NC or HRT
2020	Zhao SF, et al.	Frozen Embryo Transfer	Blank	Cleavage	The day before ET	500 IU	500 µL	1 IU/µL	48	32.38 ± 3.91	3.44 ± 0.58	55	32.96 ± 3.60	3.31 ± 0.54	NC or HRT
2021	Ji XY, et al.	Frozen Embryo Transfer	Blank	Cleavage & Blastocyst	ET Day	500 IU	< 100 µL	> 5 IU/µL	80	32.91 ± 4.79	Not mention	138	33.71 ± 5.43	Not mention	Not mention
2021	Li R, et al.	Frozen Embryo Transfer	Blank	Cleavage & Blastocyst	The day before ET	2000 IU	1000 µL	2 IU/µL	196	36.2 ± 5.1	3.1 ± 0.0	187	35.9 ± 4.4	3.2 ± 0.1	NC or HRT
2021	Xiong YL, et al.	Frozen Embryo Transfer	Blank	Blastocyst	The day before ET	500 IU	500 µL	1 IU/µL	52	32.71 ± 4.21	3.85 ± 1.07	108	33.46 ± 4.24	3.75 ± 0.84	NC or HRT
2022	Li J, et al.	Frozen Embryo Transfer	Blank	Cleavage & Blastocyst	The day before ET	2000 IU	Not mention	Not mention	32	32.3 ± 3.10	Not mention	40	31.68 ± 3.01	Not mention	NC or HRT
2022	Liu N, et al.	Frozen Embryo Transfer	Placebo	Cleavage	ET Day	1000 IU	1000 µL	1 IU/µL	51	30.26 ± 2.61	4.03 ± 0.48	50	29.75 ± 2.87	3.95 ± 0.47	NC
2022	Cheng LL, et al.	Frozen Embryo Transfer	Blank	Cleavage	3 + days pre-ET	500 IU	500 µL	1 IU/µL	44	31.30 ± 4.34	3.18 ± 0.39	36	31.33 ± 4.17	3.22士0.42	HRT
2022	Torky H, et al.	Fresh Embryo Transfer	Placebo	Blastocyst	3 + days pre-ET	500 IU	1000 µL	0.5 IU/µL	49	35.33 ± 5.11	4.43 ± 0.94	48	35.17 ± 4.23	3.67 ± 0.81	COS
2023	Xu DJ, et al.	Frozen Embryo Transfer	Placebo	Not mention	3 + days pre-ET	1000 IU	1000 µL	1 IU/µL	59	30.23 ± 2.36	6.52 ± 0.58	57	30.51 ± 2.35	6.75 ± 0.66	NC or HRT

### Risk of bias assessment

**Fig. 2:**
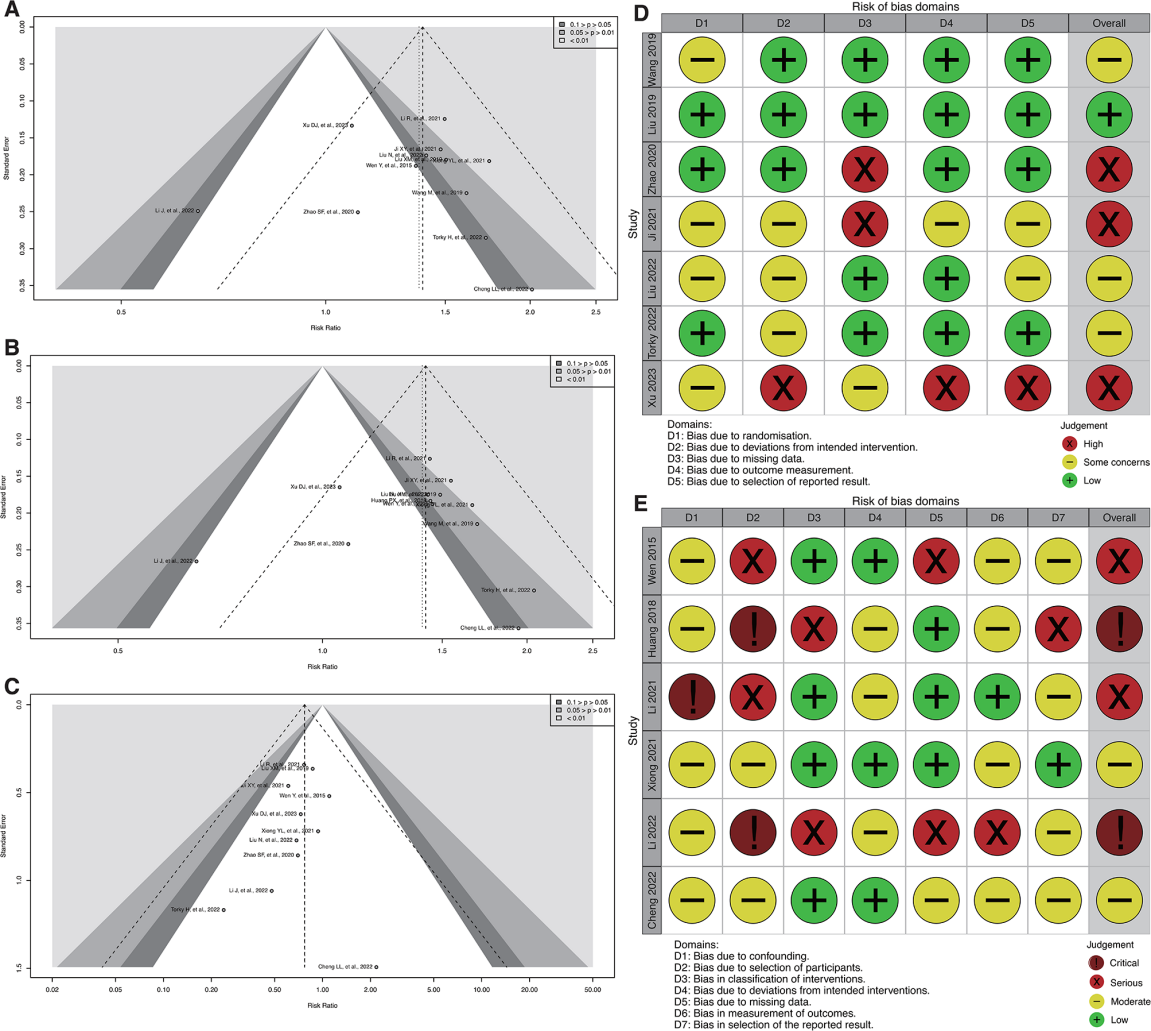
Bias Assessment and Publication Bias in Meta-Analysis Studies. (**A**, **B**, and **C**) are funnel plots for embryo implantation rate, clinical pregnancy rate, and miscarriage rate studies, respectively. (**D**) shows a traffic light plot using the RoB2 tool for randomized controlled trials (RCTs) and prospective studies. (**E**) presents a traffic light plot using the ROBINS-I tool for non-randomized control studies

In our meta-analysis, the risk of bias across all included studies was comprehensively evaluated using the GRADE approach. The retrospective studies, totaling six, were assessed using the ROBINS-I tool, while one RCT and six prospective studies were evaluated with the RoB2 tool. The results of these assessments are visually presented in Fig. 2. Additionally, methodological biases in the retrospective studies were further evaluated using the NOS, with results tabulated in Table 3. All studies assessed with the NOS scored 4 or higher, indicating a satisfactory level of quality with minimal risk of bias.

**Table 3:** Bias of retrospective research included in the analysis based on the newcastle-ottawa scale

Reference	Selection					Outcome			
	Representative	Selection	Ascertainment of exposure	Demonstration	Comparability	Outcome	Follow-up	Adequacy follow-up	Overall
Wen et al., 2015 [19]	*	-	*	*	-	*	-	-	4
Huang et al., 2018 [20]	*	-	-	*	*	*	*	*	6
Li et al., 2021 [25]	*	-	*	*	*	*	*	*	7
Xiong et al., 2021 [26]	*	*	*	*	*	*	*	*	8
Li et al., 2022 [28]	*	-	-	*	*	-	*	-	4
Cheng et al., 2022 [27]	*	*	*	*	-	*	*	-	6

Furthermore, publication bias for key outcomes such as implantation rate, clinical pregnancy rate, and miscarriage rate was analyzed using funnel plots (Fig. 2) and the Egger test (detailed in the Appendix). The findings from these analyses indicated no significant bias, as all studies included in the Egger test yielded P-values greater than 0.1. These rigorous bias assessments ensure the reliability and credibility of the findings presented in our meta-analysis, providing reproductive medicine professionals with robust evidence on the effectiveness of the interventions studied.

### Primary outcomes

**Fig. 3:**
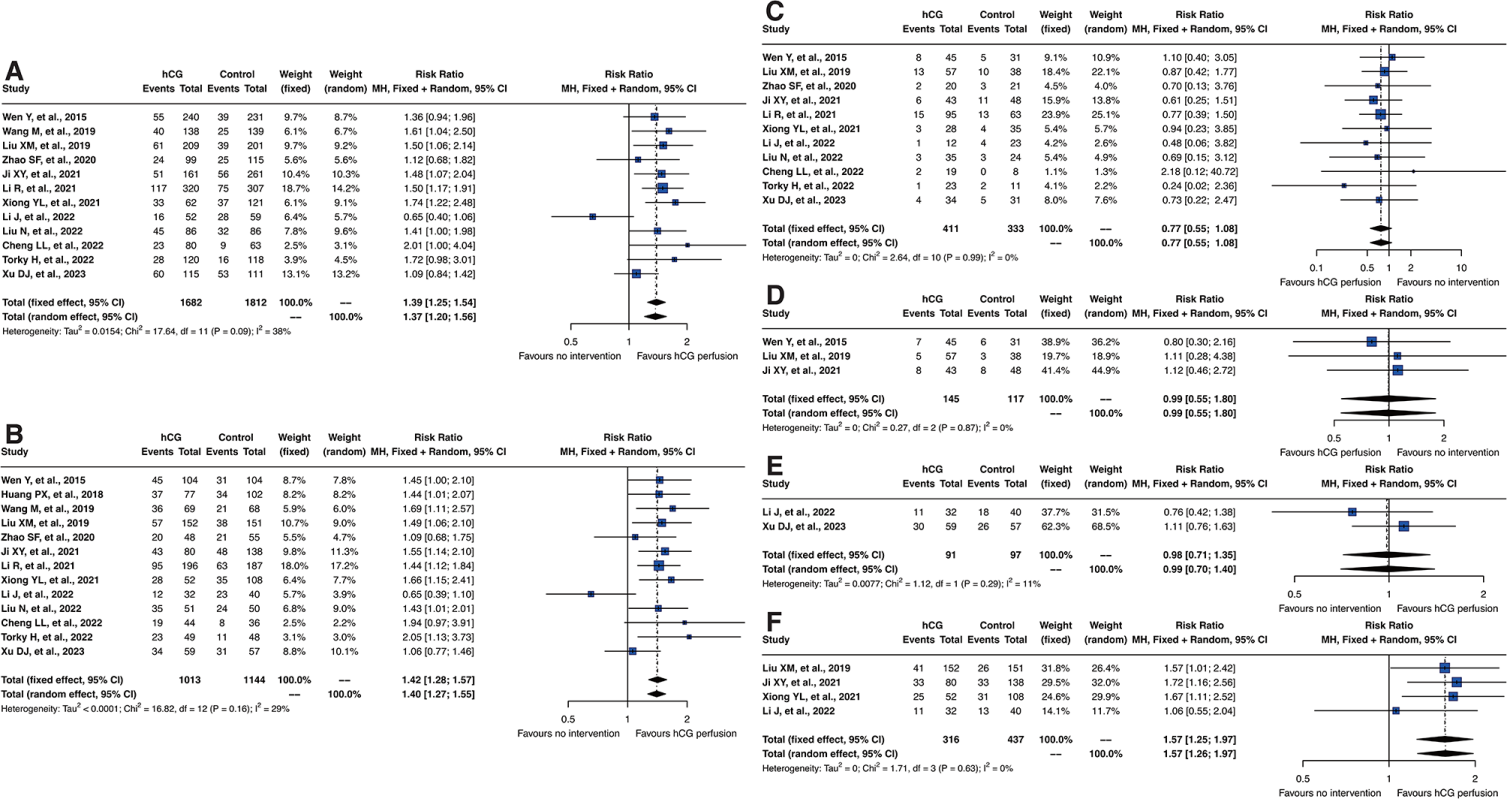
Forest Plots of Clinical Outcomes from the Included Studies. (**A**) Forest plot for embryo implantation rate, (**B**) clinical pregnancy rate, (**C**) miscarriage rate, (**D**) ectopic pregnancy rate, (**E**) ongoing pregnancy rate, and (**F**) live birth rate

The primary outcomes of this meta-analysis focused on assessing the efficacy and safety of intrauterine hCG perfusion in patients with recurrent implantation failure. The key findings, summarized in Fig. 3, reflect the therapeutic benefits and safety profile of hCG treatment. The results, presented through RR with 95% CI, provide a comprehensive understanding of hCG’s role in improving clinical outcomes in these patients.

#### Embryo implantation rate

Analysis of embryo implantation rates from 12 included studies demonstrated a clear improvement with hCG perfusion. The fixed model RR for embryo implantation was 1.39 [95% CI: 1.25; 1.54], and the random model RR was 1.37 [95% CI: 1.20; 1.56]. These results signify a significant benefit for patients with a history of three or more implantation failures.

#### Clinical pregnancy rate

All 13 studies provided data on clinical pregnancy rates. The analysis indicated a significant improvement in these rates with hCG treatment, evident in both fixed effect and random effect models. The fixed model RR was 1.42 [95% CI: 1.28; 1.57] and random model RR was 1.40 [95% CI: 1.27; 1.55], highlighting the efficacy of hCG in enhancing clinical pregnancy rates.

#### Miscarriage rate

In our analysis, 11 studies provided data on miscarriage rates. Although hCG treatment did not demonstrate a statistically significant reduction in miscarriage rates, both the fixed model and the random model showed a RR of 0.77 [95% CI: 0.55; 1.08]. This result, while not reaching statistical significance, suggests a trend toward decreased miscarriage occurrences in the hCG group. The forest plot further illustrated this positive trend, supporting the potential beneficial effect of hCG treatment in reducing miscarriages.

#### Safety and multiple pregnancy rate

Concerning safety, particularly the rate of multiple pregnancies, hCG perfusion did not show an increase. The results from three studies included in this analysis confirmed that the safety profile of hCG treatment was within acceptable parameters, underscoring its safety in clinical applications without substantially increasing the risk of multiple pregnancies. However, due to the limited number of studies included, further in-depth research and exploration are necessary to comprehensively assess the long-term safety and effectiveness of hCG perfusion. This will ensure robust evidence-based practices in reproductive medicine.

#### Ongoing and live birth rate

The ongoing pregnancy rate was evaluated in two studies, while data on live births were available from four studies. The analysis indicated no significant differences in ongoing pregnancy rates. This outcome supports the safety of intrauterine hCG perfusion; however, the limited number of studies included necessitates further investigation. Continued research is essential to conclusively establish the safety profile of hCG perfusion throughout the entire pregnancy process. This will help to ensure that the treatment’s efficacy and safety are adequately documented and understood in clinical applications. Regarding the live birth rate, after a comprehensive analysis, the fixed model RR was determined to be 1.57 [95% CI: 1.25; 1.97], and the random model RR was similarly 1.57 [95% CI: 1.26; 1.97]. These results highlight the beneficial impact of intrauterine hCG perfusion on increasing live birth rates, demonstrating its efficacy in enhancing successful pregnancy outcomes.

### Dosage-dependent efficacy of intrauterine hCG perfusion

**Fig. 4:**
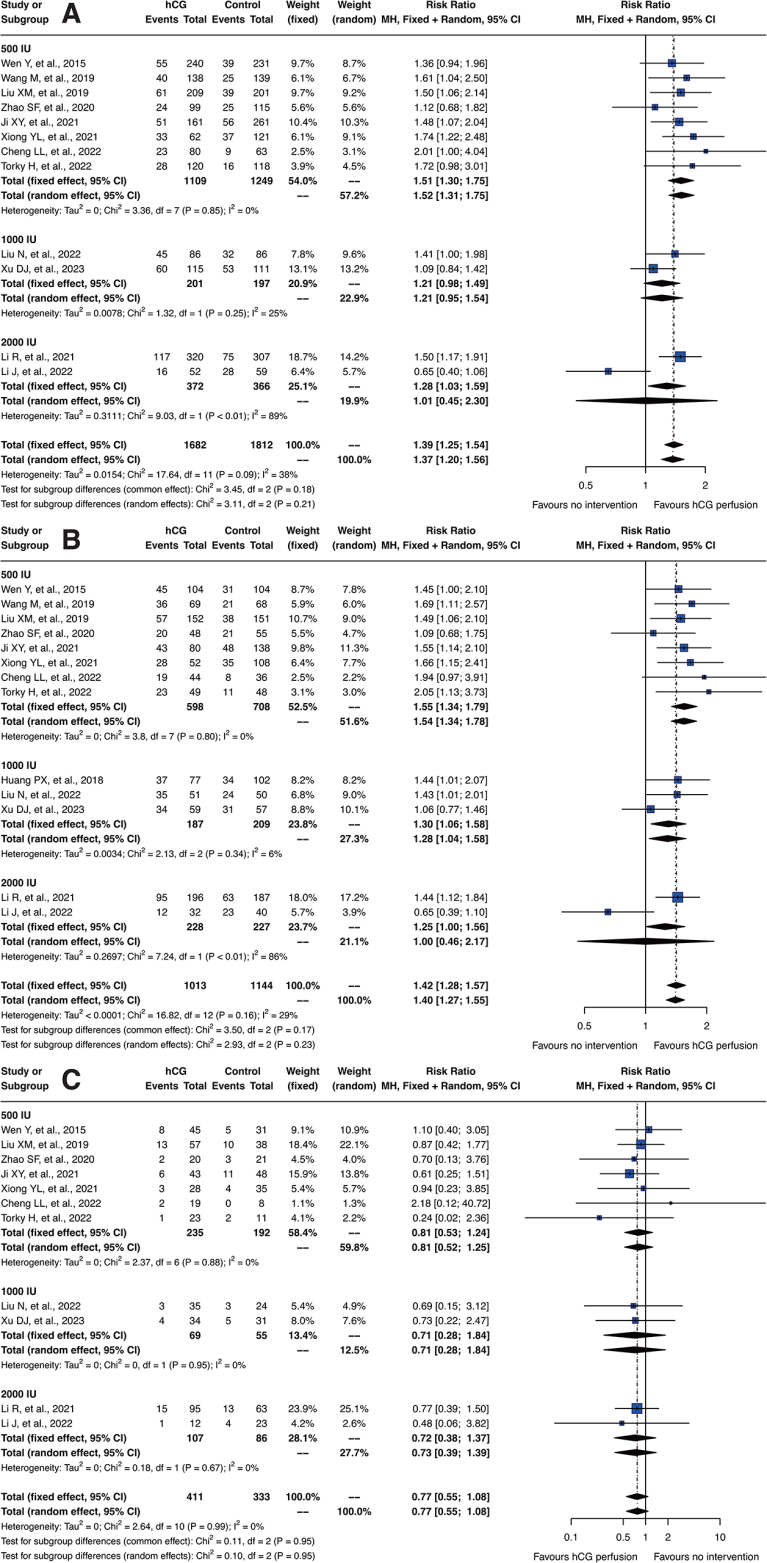
Subgroup analysis of hCG dosage effects on reproductive outcomes. Forest plots depicting the effects of varying hCG dosages on (**A**) embryo implantation rates, (**B**) clinical pregnancy rates, and (**C**) miscarriage rates

In our meta-analysis, we conducted detailed subgroup analyses of hCG dosages on embryo implantation rates, clinical pregnancy rates, and miscarriage rates, as depicted in Fig. 4. The dosages assessed included 500 IU, 1000 IU, and 2000 IU. The results demonstrated a clear advantage for the 500 IU dosage across various outcomes. Specifically, the RR for embryo implantation rates in the fixed effect model was 1.51 [95% CI: 1.30; 1.75] for 500 IU, significantly higher compared to 1000 IU which posted an RR of 1.21 [95% CI: 0.98; 1.49]. In terms of clinical pregnancy rates, the fixed effect model showed an RR of 1.55 [95% CI: 1.34; 1.79] for 500 IU, again outperforming the 1000 IU dosage, which had an RR of 1.30 [95% CI: 1.06; 1.58]. For miscarriage rates, there were no significant differences noted among the three dosage groups, although they exhibited similar trends.

It is important to emphasize that, although only two studies involved the 2000 IU dosage, they produced completely contradictory trends. Similarly, the studies involving the 1000 IU dosage, limited to only three, also present uncertainties due to their small number and mixed outcomes. This discrepancy highlights the uncertainty surrounding the effectiveness and potential side effects of higher dosages. Given the potential for bias introduced by the small sample sizes and conflicting results for both 1000 IU and 2000 IU dosages, the conclusions regarding these dosages should be interpreted with caution. The limited data available may not fully represent the true effects of these higher dosages, and thus, any conclusions drawn from these findings must be approached judiciously.

### Deeper investigation on hCG volume and Concentration effects

**Fig. 5:**
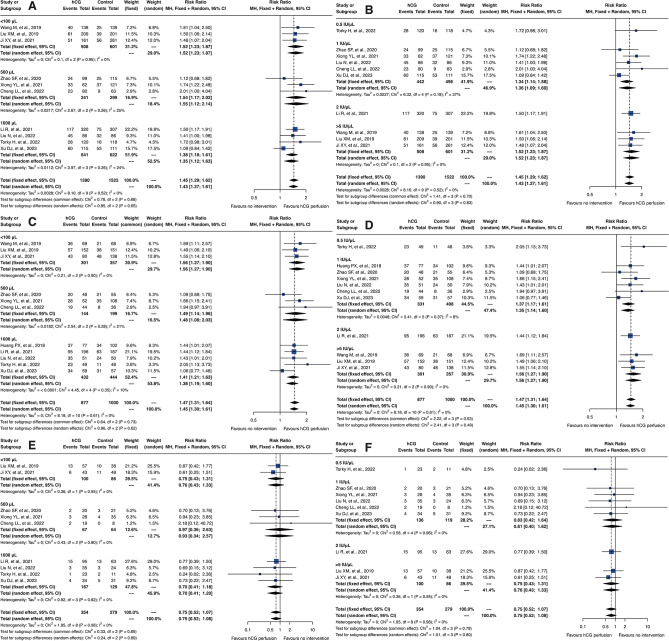
Impact of hCG perfusion volume and concentration on reproductive outcomes. Forest plots demonstrating the effects of different volumes and concentrations of hCG perfusion on reproductive outcomes. (**A**, **C** and **E**) represent the effects of varying perfusion volumes on embryo implantation rates, clinical pregnancy rates, and miscarriage rates, respectively. (**B**, **D**, and **F**) illustrate the impacts of different hCG concentrations on the same outcomes

Furthermore, we conducted additional subgroup analyses focusing on the volume of fluid used and the final concentration of hCG, detailed in Fig. 5. The results indicated that smaller fluid volumes and higher hCG concentrations were associated with better clinical outcomes. Specifically, in the fixed effect model, the RRs for embryo implantation and clinical pregnancy rates were more favorable at lower volumes and higher concentrations. For volumes of 500µL and concentrations of 2 IU/µL, the RR for embryo implantation was 1.54 [95% CI: 1.17; 2.02] and 1.50 [95% CI: 1.17; 1.91], respectively. Similarly, volumes less than 100µL and concentrations over 5 IU/µL both showed an RR of 1.52 [95% CI: 1.23; 1.87]. For clinical pregnancy rates, the analysis produced RRs of 1.49 [95% CI: 1.14; 1.96] for 500µL and 1.44 [95% CI: 1.12; 1.84] for concentrations of 2 IU/µL; while for volumes less than 100µL and concentrations over 5 IU/µL, the RRs were consistently 1.56 [95% CI: 1.27; 1.90].

In terms of miscarriage rates, no significant differences were observed between different perfusion volumes and hCG concentrations. However, given the small number of studies in each subgroup, caution should be exercised in interpreting these results and in making clinical recommendations. This suggests that while smaller volumes and higher concentrations of hCG may enhance implantation and pregnancy rates, the findings should be validated with further research to substantiate these trends and inform clinical practice more definitively.

### Impact of hCG perfusion timing and transfer type on clinical outcomes

**Fig. 6:**
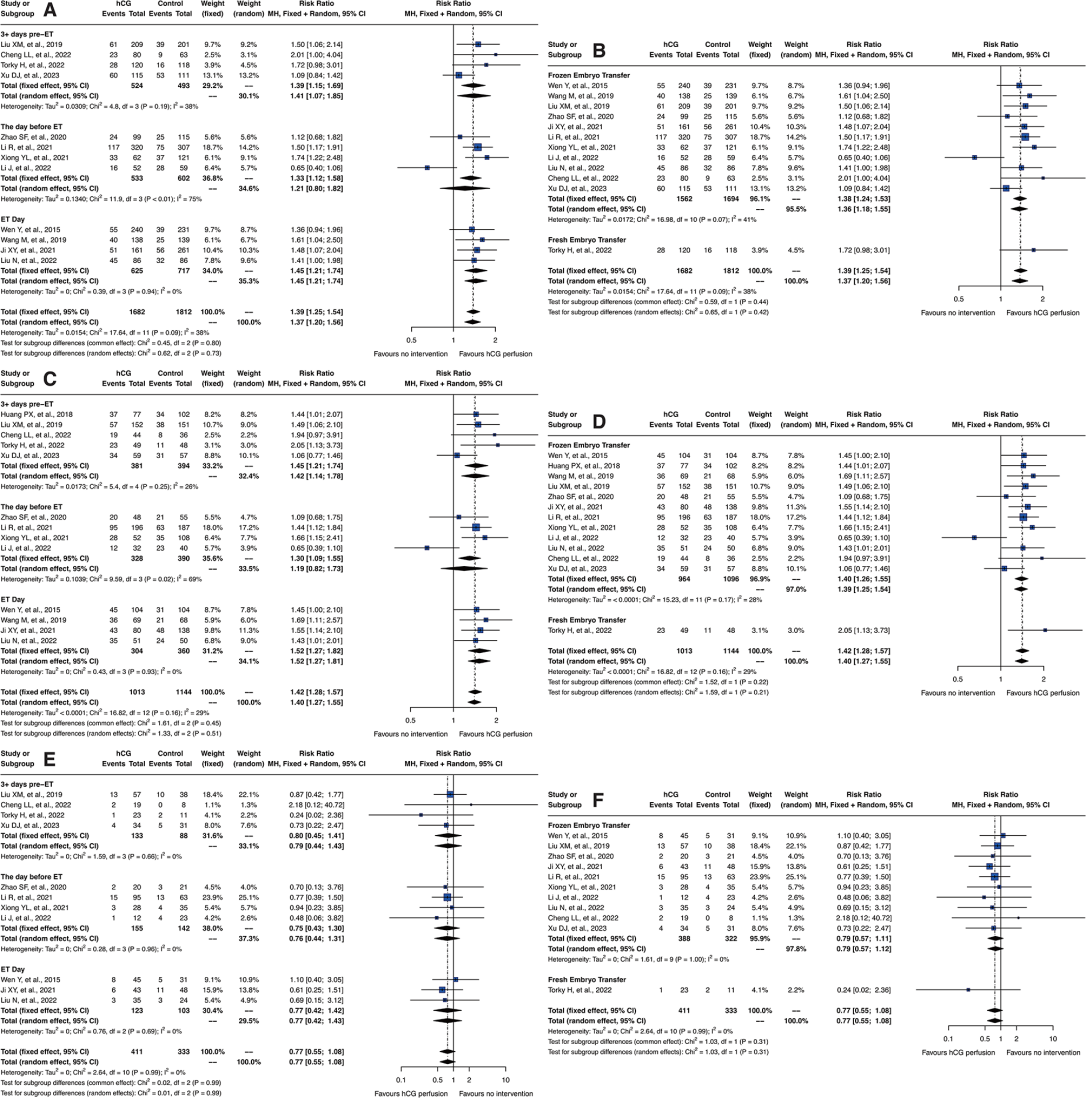
Effects of hCG perfusion timing and transfer type on reproductive outcomes. Forest plots illustrating the impact of hCG perfusion timing and embryo transfer type (fresh vs. FET) on reproductive outcomes. (**A**, **C** and **E**) display the effects of different hCG perfusion timings on embryo implantation rates, clinical pregnancy rates, and miscarriage rates, respectively. (**B**, **D** and **F**) show the impacts of fresh transfers and frozen embryo transfers (FET) on these same outcomes

In the comprehensive subgroup analyses, which are detailed in Fig. 6, we evaluated the effects of hCG perfusion based on the timing of administration and the type of embryo transfer (FET vs. fresh ET). For the timing of hCG administration, the analysis encompassed three primary intervals: more than three days before embryo transfer, one day before, and on the day of transfer.

The results indicate significant benefits in both embryo implantation rates and clinical pregnancy rates, regardless of the timing of administration or the type of embryo transfer. Moreover, hCG intrauterine perfusion exhibited a safe profile concerning miscarriage rates across all timing and transfer scenarios. This demonstrates hCG’s crucial role in modulating the uterine environment, enhancing conditions favorable for embryo implantation and effective maternal-fetal interaction, thereby improving overall pregnancy outcomes in patients with recurrent implantation failure.

However, it is important to note that there is a distinct lack of clinical studies focusing on hCG perfusion in fresh embryo transfers. The existing data predominantly relate to frozen embryo transfers, and while the outcomes are positive, the effectiveness and safety of hCG perfusion during fresh transfer cycles remain less explored. This gap underscores the need for cautious interpretation of hCG perfusion’s benefits in fresh transfers and highlights an urgent need for more research in this specific area to better inform clinical practices.

### Analysis of control types in hCG perfusion studies

**Fig. 7:**
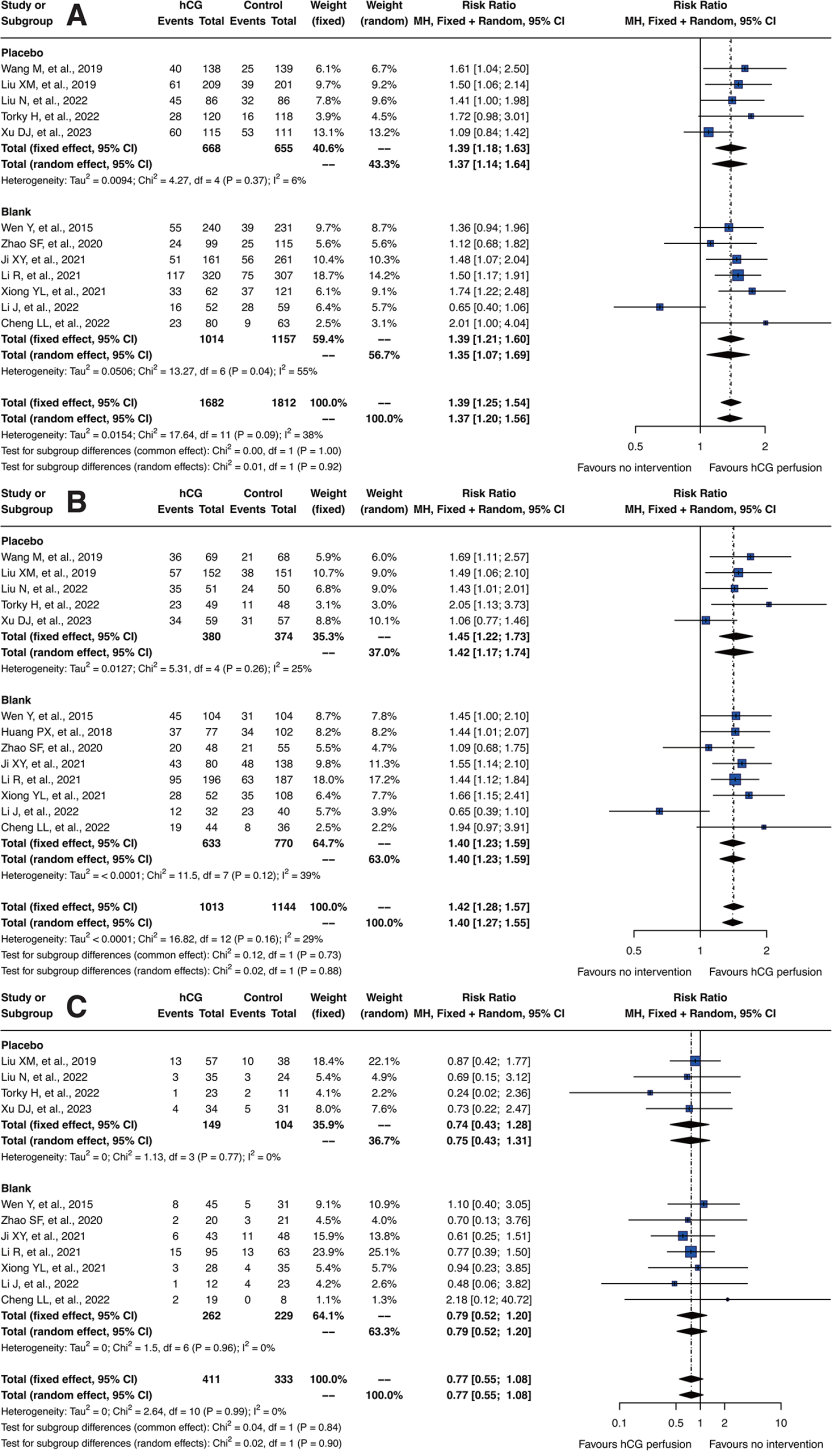
Forest plots analyzing differences in reproductive outcomes by control types. Forest plots comparing the effects of different control types on reproductive outcomes. (**A**) shows differences in embryo implantation rates, (**B**) illustrates clinical pregnancy rates, and (**C**) details miscarriage rates across various control groups

The subgroup analyses were also conducted based on the type of control used, specifically comparing outcomes between placebo and blank controls (Fig. 7). This detailed analysis focused on embryo implantation rates, clinical pregnancy rates, and miscarriage rates. The results revealed that irrespective of the control type, the outcomes were consistent with the overall findings of the study, indicating that intrauterine hCG perfusion consistently enhances both the effectiveness and safety of clinical outcomes.

However, it is important to note that the design of the control groups often varied due to differences in study protocols, which introduces some limitations to the subgroup analyses. Nonetheless, the uniform results across different control types further substantiate the efficacy of hCG perfusion. This consistency across various study designs not only reinforces the benefits of hCG treatment but also underscores its potential utility in improving reproductive success in clinical settings.

### Analysis of embryonic development stages in hCG perfusion

**Fig. 8:**
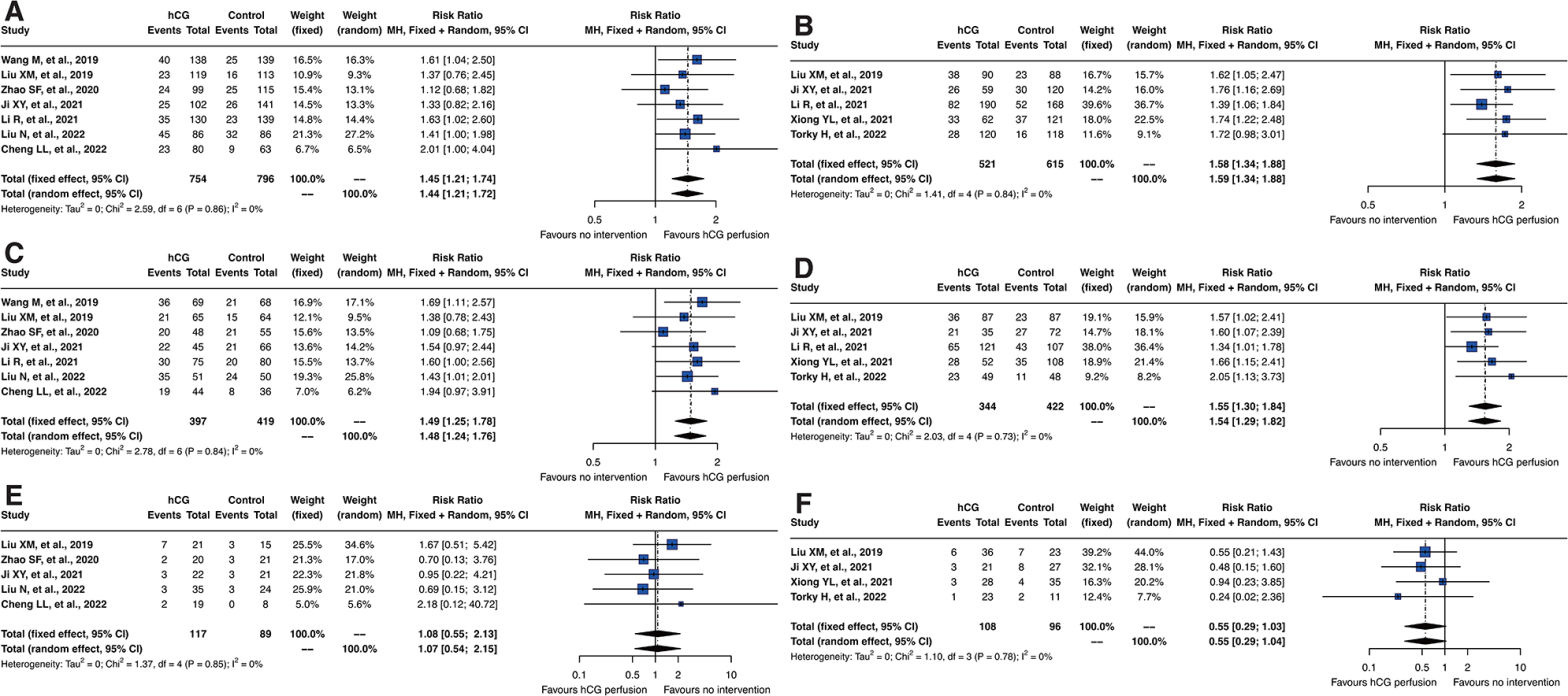
Forest plots of reproductive outcomes by embryo developmental stages. Forest plots comparing the effects of embryo developmental stages on reproductive outcomes. (**A**, **C** and **E**) illustrate the impacts on embryo implantation rates, clinical pregnancy rates, and miscarriage rates for cleavage-stage embryos, respectively. (**B**, **D** and **F**) detail these outcomes for blastocyst-stage embryos, providing a visual comparison of efficacy between the two stages

Further detailed subgroup analyses were conducted on the types of embryos transferred, as illustrated in Fig. 8. This analysis specifically examined embryo implantation rates, clinical pregnancy rates, and miscarriage rates for both cleavage-stage embryos and blastocyst transfers. The results indicated that both types of embryo transfers—cleavage-stage and blastocyst—exhibited RR values similar to the overall findings. This consistency demonstrates that intrauterine hCG perfusion significantly enhances outcomes irrespective of the embryo stage, offering substantial benefits in cases of RIF. This evidence supports the broad applicability of hCG perfusion across different embryonic stages, reinforcing its role as a critical intervention to improve reproductive success in diverse clinical scenarios.

## Discussion

### Summary of key findings

This meta-analysis comprehensively assessed thirteen studies, comprising six retrospective and six prospective studies from single centers, along with one multi-center RCT totaling 2,157 participants. It revealed that intrauterine hCG perfusion plays a crucial role in enhancing embryo implantation and clinical pregnancy rates in patients with RIF, while maintaining clinical safety. Subgroup analyses further indicated that a 500 IU dosage is sufficient to improve clinical outcomes in RIF, and that smaller perfusion volumes (up to a maximum of 500µL) combined with higher concentrations (at least 2 IU/µL) may lead to even better outcomes. Significant improvements in clinical outcomes were observed regardless of the timing of administration, the type of embryos transferred, whether fresh or frozen.

However, it is important to note that the analyses in this meta-analysis involved a limited number of studies, including only one randomized controlled trial alongside several retrospective and prospective studies. This distribution necessitates cautious interpretation and application of these findings, as the evidence level varies significantly across the different study types. Consequently, more research is needed to confirm these results and ensure that recommendations are based on robust evidence. Despite these limitations, this comprehensive evaluation confirms the probably efficacy and safety of hCG perfusion, highlighting its advantages in terms of affordability and accessibility. These attributes make hCG perfusion particularly valuable in economically less developed regions, offering a feasible alternative to more expensive treatments like GH, ERA, and various immunotherapies, which may be less available.

### Context and comparison with other treatments

The application of hCG for intrauterine perfusion in treating RIF stands out primarily due to its significant biological advantages and its excellent safety profile. hCG, a hormone naturally secreted by the embryonic trophoblast cells, plays a crucial role in maintaining early pregnancy. Its use in treatment mimics this natural role, providing reassurance about its safety. This natural origin of hCG ensures that it is well-tolerated and minimizes the risk of adverse reactions typically associated with synthetic drugs.

Biologically, hCG protects endometrial stromal cells from apoptosis induced by oxidative stress and effectively modulates the immune system to support pregnancy [33]. This includes crucial processes such as the induction and differentiation of regulatory T cells, suppression of effector T lymphocytes, and the regulation of macrophage migration and uterine natural killer cell activity [34–36]. These mechanisms are vital for enhancing embryonic differentiation, improving endometrial receptivity, and facilitating maternal-fetal immune tolerance [37], all of which are essential for successful embryo implantation and pregnancy continuation.

Moreover, hCG’s role extends beyond biological effects to provide logistical and economic benefits. It is a cost-efficient option that is readily procurable, making it accessible for a broad range of patients. This accessibility is particularly important in reducing the financial burden of fertility treatments on patients. The combination of hCG’s biological importance, its safety derived from its natural role in pregnancy, and economic advantages underscore its value as a superior treatment modality in assisted reproductive technology.

Treating RIF involves a multifaceted approach, focusing on enhancing endometrial receptivity and modulating the immune system. While GH is noted for its potential to upregulate factors like VEGF and IGF-1, improving subendometrial blood flow and the uterine environment, the evidence supporting its routine use remains uncertain [38, 39]. Similarly, the ERA utilizes transcriptomic profiling to identify the optimal window for implantation, though its efficacy in improving clinical outcomes continues to be evaluated [40].

Transitioning from hormonal and diagnostic approaches to immune-based therapies, a variety of immunotherapies such as IVIG and PBMC perfusion have been proposed to enhance the endometrial environment conducive to implantation. However, despite their theoretical benefits, these interventions lack conclusive evidence and remain in the experimental stages [41, 42]. IVIG is believed to beneficially alter immune responses, and PBMC perfusion is thought to improve local immune conditions within the endometrium, yet both require more robust clinical validation [43, 44].

Further, other immunomodulatory therapies like G-CSF and PRP are utilized for their potential to release growth factors and cytokines, crucial for embryo implantation. However, similar to IVIG and PBMC, the definitive benefits of G-CSF and PRP in the context of RIF treatment have not been conclusively established and continue to be topics of active research [45–48].

In comparison, the use of intrauterine hCG perfusion stands out not only for its ability to significantly improve clinical pregnancy and embryo implantation rates but also for its established safety profile. The clearer and more consistent evidence supporting hCG’s effectiveness in clinical settings offers a compelling alternative to the more uncertain or experimental outcomes associated with GH, ERA, and various immunotherapies. This meta-analysis underscores hCG’s prominence as a particularly valuable intervention in the arsenal of assisted reproductive technologies.

### Direct vs. systemic effects of hCG on endometrial environment

While the efficacy of intrauterine hCG perfusion in enhancing embryo implantation is well-documented [7, 13, 49], questions remain about the necessity of its action directly within the endometrium. hCG is traditionally administered systemically to support the luteal phase in ART [50]. These systemic applications raise considerations about whether direct intrauterine administration offers additional benefits.

Systemic administration of hCG, typically through injections, is known to stimulate the ovaries and support the corpus luteum, which in turn secretes progesterone vital for maintaining the early stages of pregnancy [51]. This systemic approach indirectly affects the endometrium by increasing progesterone levels, which enhances endometrial receptivity to an implanting embryo. However, direct intrauterine administration of hCG may influence the endometrial environment more directly and immediately.

Research suggests that intrauterine hCG perfusion can lead to a more localized and potent modification of the endometrial immune environment and enhance the expression of factors directly involved in mediating implantation [52]. For example, direct application of hCG to the endometrium is thought to increase the local concentration of cytokines, growth factors, and other molecules critical for successful implantation that systemic administration may not sufficiently impact [53]. This localized approach ensures that hCG is present at the site of implantation at optimal concentrations to exert its effects on the endometrial stromal cells, immune cells, and angiogenic factors.

Additionally, intrauterine hCG application has been shown to have direct effects on the endometrium, such as enhancing the secretion of LIF and VEGF [54, 55], which are crucial for the implantation process. These direct endometrial actions suggest that intrauterine delivery of hCG may be more effective than systemic administration in cases of repeated implantation failure, where local deficiencies in these implantation factors may exist.

Therefore, while systemic hCG injections indirectly affect the endometrial environment, intrauterine hCG administration offers a more targeted approach, potentially enhancing embryo implantation. This distinct impact underscores the necessity for comparative studies to establish the most effective administration routes for hCG, particularly for patients who do not respond to conventional treatments. To further refine treatment strategies, assessing the endometrial immune profile and decidualization score before hCG administration could be invaluable. This evaluation would allow clinicians to tailor interventions more precisely to individual endometrial conditions, improving outcomes in challenging cases like repeated implantation failure. Thus, integrating routine assessments of the endometrial environment could significantly enhance the personalization and effectiveness of fertility treatments.

### Implications for practice and research

Given the uncertainties surrounding conventional interventions, intrauterine perfusion of hCG presents a promising alternative. The use of hCG to improve clinical pregnancy rates in patients with RIF could potentially offer a more reliable option, bolstered by emerging research that underscores its role in enhancing endometrial receptivity [52].

Recent advancements in the landscape of IVF treatments, including the adoption of the freeze-all strategy, highlight a shift towards improving endometrial receptivity, partly to mitigate the negative effects of ovarian stimulation on the endometrium [56, 57]. Evidence suggests that transferring frozen embryos, detached from the ovarian stimulation cycle, can yield higher pregnancy rates, emphasizing the crucial role of endometrial receptivity in the success of IVF treatments [58]. The well-documented biological role of hCG in facilitating implantation aligns with these findings [59]. While some studies, such as those aligned with the Cochrane review, suggest no significant benefit of hCG for IVF outcomes [60], others report favorable outcomes, particularly in terms of implantation, clinical pregnancy, and ongoing pregnancy rates [49]. These mixed results suggest that while hCG’s benefits are clear in some contexts, its variable impact reflects the need for further study to clarify its role.

In clinical settings, the strategic timing and precise dosage of intrauterine hCG perfusion are crucial for enhancing implantation success, particularly in patients with a history of RIF. Although some studies recognize the benefits of hCG perfusion, significant debate persists over the optimal timing and dosage [61]. Research and current guidelines suggest that administering hCG shortly before embryo transfer can significantly improve outcomes by ensuring higher concentrations of hCG are present at the site of action [62], potentially overcoming barriers related to suboptimal endogenous LH activity or inadequate endometrial responsiveness.

The dosage of intrauterine hCG perfusion in IVF treatments lacks standardization, with effectiveness proving to be dosage-dependent [61]. Studies show that lower dosages, less than 500 IU, typically do not enhance live birth rates, whereas higher dosages of 500 IU or more may improve outcomes [63]. However, the impact of hCG perfusion varies with the stage of embryo development at transfer, underscoring the importance of personalized hCG dosing strategies [63]. These strategies should consider the specific timing of the embryo transfer and adapt to the unique endometrial and physiological conditions of each patient to optimize the effectiveness of treatment.

Given these insights, it is advisable for clinicians to systematically integrate hCG perfusion into RIF treatment protocols, especially in scenarios involving frozen embryo transfers where synchronization of endometrial receptivity with the embryo’s developmental stage is critical. Adjusting both the timing and dosage of hCG perfusion according to individual needs and response patterns could provide a more personalized approach to treatment, potentially improving clinical outcomes significantly.

For future research, there is a compelling need to explore the differential responses to hCG treatment among various patient groups, such as those with RIF compared to women of reproductive age, and to assess the distinct effects of hCG in fresh versus frozen embryo transfers. This involves conducting large-scale, multi-center RCTs to determine the optimal dosage and timing of hCG administration. Additionally, from the perspective of basic medical research, it is crucial to investigate the specific effects of hCG on endometrial stromal cells and embryos. This includes a thorough examination of the impact of varying doses and timing of hCG on endometrial protein expression and related pathways, particularly in patients with RIF. Consideration of the outcomes of continuous versus single-dose hCG administration, as well as its influence on uterine contractions and endocrine mechanisms, are also important areas of study. These research directions are essential for a more comprehensive understanding of the biological actions and mechanisms of hCG, and will help to tailor treatment approaches based on specific patient characteristics to enhance therapeutic efficacy and safety, addressing the complexities of RIF with a more individualized treatment protocol.

These enhanced research and clinical strategies will not only contribute to the scientific community’s knowledge but also potentially lead to more effective and safer treatment modalities, reflecting the practical implications of current evidence while paving the way for future advancements.

### Strengths and limitations

Our meta-analysis builds on findings from the 2022 study by Bede Tyler et al., which reported significant improvements in clinical pregnancy rates with hCG supplementation (RR 1.232, 95% CI 1.099–1.382) in a general assisted reproduction context [64]. While corroborating the potential of hCG to enhance clinical pregnancy outcomes, our study extends these insights to the specific challenges faced by patients with RIF, highlighting nuanced benefits in both clinical pregnancy and, tentatively, live birth rates. Despite the positive findings in our meta-analysis, several limitations must be acknowledged that could impact the interpretation and generalizability of the results. The limited sample size and lack of diversity among the studied populations restrict the generalizability of our findings, which may influence the applicability of results across different demographic and geographic groups. Variability in endometrial preparation protocols among the studies introduces further limitations, as inconsistencies in these procedures can affect outcome comparability and clinical relevance. Additionally, potential biases inherent in the design and methodology of the included studies complicate the interpretation of results, arising from the specific methods employed in conducting and reporting research.

The absence of long-term follow-up data is a significant drawback as such data are crucial for understanding the durability of treatment effects and identifying any potential delayed side effects associated with hCG treatment. Additionally, there is an inadequate understanding of the mechanisms through which hCG enhances endometrial receptivity and supports embryo implantation, highlighting the need for more in-depth biological and mechanistic studies.

There remains substantial debate regarding the optimal timing and dosage for hCG administration. Although our findings suggest that a dose of 500 IU can significantly improve outcomes, establishing a standardized approach requires further research. The studies included in our meta-analysis vary widely regarding age groups, definitions of RIF, inclusion and exclusion criteria, endometrial preparation protocols, embryo stages, embryo quality, and the number of embryos transferred. This heterogeneity, along with individual patient differences, introduces variability in outcomes and poses challenges in drawing uniform conclusions.

To effectively address these limitations, it is imperative to conduct larger-scale, multi-center randomized controlled trials. These trials should not only aim to standardize and control the variables but also focus on tailoring treatments based on specific patient characteristics to enhance therapeutic efficacy and safety. Such comprehensive studies will help refine our understanding of hCG’s role in treating RIF, leading to improved treatment protocols and outcomes in reproductive medicine. These efforts are vital for advancing scientific knowledge and achieving better clinical practices.

## Conclusions

This meta-analysis, comprising six retrospective and six prospective studies from single centers, along with one multi-center RCT totaling 2,157 participants, indicates that intrauterine hCG perfusion probably enhances embryo implantation, clinical pregnancy rates, and live birth rates slightly in patients with RIF. Evidence further suggests that a dosage of 500 IU and a maximum volume of 500µL, with concentrations of at least 2 IU/µL, might be linked to these potential improvements, with possible benefits observed across various timings and types of embryo transfers.

These findings should be interpreted with considerable caution due to the substantial heterogeneity primarily arising from the differences in study types, as well as other limitations inherent to observational research. The preliminary nature of these results, particularly concerning live birth rates which are based on a limited number of studies, necessitates cautious interpretation and further discussion. Given the critical importance of live birth rates, along with the need to improve clinical pregnancy and implantation rates, more rigorously designed RCTs are essential to more definitively assess the efficacy and safety of intrauterine hCG perfusion.

### Electronic supplementary material

Below is the link to the electronic supplementary material.

Supplementary Material 1

Supplementary Material 2

## Data Availability

No datasets were generated or analysed during the current study.

## Abbreviations

RIF: Recurrent Implantation Failure
ART: Assisted Reproductive Technology
IVF: In Vitro Fertilization
ESHRE: European Society Of Human Reproduction And Embryology
hCG: Human Chorionic Gonadotropin
PRISMA: Preferred Reporting Items for Systematic Reviews and Meta-Analyses
MeSH: Medical Subject Headings
ICSI: Intracytoplasmic Sperm Injection
NOS: Newcastle-Ottawa Scale
GRADE: Grading of Recommendations Assessment, Development and Evaluation
RR: Relative Risk CI: Confidence Intervals
ET: Embryo Transfer
FET: Frozen Embryo Transfer
RCT: Randomized Controlled Trial
GH: Growth Hormone
VEGF: Vascular Endothelial Growth Factor
IGF-1: Insulin-Like Growth Factor 1
ERA: Endometrial Receptivity Array
IVIG: Intravenous Immunoglobulin
PBMC: Peripheral Blood Mononuclear Cell
G-CSF: Granulocyte Colony-Stimulating Factor
PRP: Platelet-Rich Plasma
