# Supplementary material for: Meta-analysis of intrauterine hCG perfusion efficacy in recurrent implantation failure as defined by ESHRE guidelines
Source: BMC Pregnancy Childbirth. 2024 Jul 9;24:468. doi: 10.1186/s12884-024-06662-1 (PMC11234711; doi:10.1186/s12884-024-06662-1)
Supplement: Supplementary file 1 — Supplementary Material 1 [file 12884_2024_6662_MOESM1_ESM.docx]

Supplementary Appendix for article entitled

“Meta-Analysis of Intrauterine hCG Perfusion Efficacy in Recurrent Implantation Failure as Defined by ESHRE Guidelines.

**Contents**

[1. Search Strategy 2](#_Toc168756225)

[1.1. PubMed and MEDLINE 3](#_Toc168756226)

[1.2. Embase 3](#_Toc168756227)

[1.3. Cochrane Library 4](#_Toc168756228)

[1.4. Web of Science 5](#_Toc168756229)

[1.5. Key Chinese databases 6](#_Toc168756230)

[2. Studies Included After Initial Screening" 7](#_Toc168756231)

[3. Detailed Exclusion of Non-Compliant Studies 12](#_Toc168756232)

[4. Included Studies for Meta-analysis 16](#_Toc168756233)

[5. NOS Assesment Criteria 18](#_Toc168756234)

[6. Egger Test for Publication Bias 19](#_Toc168756235)

1. Search Strategy

The search strategy is designed to retrieve scientific articles from databases using a series of specific terms grouped into five distinct searches. Here’s a breakdown of each search string and their intended use:

**Chorionic Gonadotropin and Related Terms:**

This search targets articles discussing various terms related to chorionic gonadotropin, a hormone important in pregnancy. Keywords include 'Chorionic Gonadotropin', 'chorionic', 'gonadotropin', 'choriogonadotropin', 'hCG', and 'CG'. These are searched within the titles, abstracts, or as MeSH (Medical Subject Headings) terms to capture all relevant literature.

**Uterine Factors and Actions:**

This string focuses on the uterus and related terms (e.g., 'uterus', 'uterine', 'endometrium', etc.) combined with actions performed on these structures such as 'perfusion', 'infusion', 'instillation', 'filling', and 'injection'. The aim is to find articles discussing procedures or states affecting the uterine environment.

**Reproductive Health and Procedures:**

This comprehensive search covers a broad range of topics within reproductive health and assisted reproductive technologies (ART). It includes terms like 'Embryo Transfer', 'Fertility', 'Infertility', 'Reproductive Techniques, Assisted', 'Pregnancy', 'Abortion, Spontaneous', 'Embryo Implantation', 'Sperm Injections, Intracytoplasmic', and 'Fertilization in Vitro'.

**Implantation Failures and Related Conditions:**

Here, the focus is on implantation failures and related reproductive challenges. Keywords such as 'repeated', 'multiple', 'recurrent', 'implantation', 'engraftment', 'failure', and acronyms like 'RIF' (Repeated Implantation Failure) and 'MIF' (Multiple Implantation Failures) are included to identify studies on recurrent reproductive issues.

**Combined Search:**

The final search (#5) combines all the previous searches (#1 through #4) using the logical operator AND. This is intended to find literature that intersects all the specified themes, providing a focused dataset on studies that discuss chorionic gonadotropin, uterine procedures, broader reproductive health issues, and specific challenges like implantation failures.

This strategy is structured to capture a comprehensive dataset that covers both broad topics in reproductive medicine and specific issues such as implantation failures, making it highly useful for researchers looking at intricate aspects of reproductive health and interventions.

- 1. PubMed and MEDLINE

| **#** | **Searches ("1900/01/01"[Date - Publication] : "2023/12/31"[Date - Publication])** | **Results** |
| --- | --- | --- |
| 1 | ("Chorionic Gonadotropin"[MeSH Terms] OR  "chorionic"[Title/Abstract] OR  "gonadotropin"[Title/Abstract] OR  "choriogonadotropin"[Title/Abstract] OR  "hCG"[Title/Abstract] OR CG"[Title/Abstract]) |  |
| 2 | (("uterus"[Title/Abstract] OR "uterine"[Title/Abstract] OR "endometrium"[Title/Abstract] OR "endometrial"[Title/Abstract] OR "intrauterine"[Title/Abstract] OR "myometrium"[Title/Abstract] OR "cervix"[Title/Abstract] OR "cervical"[Title/Abstract]) AND ("perfusion"[Title/Abstract] OR "infusion"[Title/Abstract] OR "instillation"[Title/Abstract] OR "filling"[Title/Abstract] OR "injection"[Title/Abstract] )) |  |
| 3 | (("Embryo Transfer"[MeSH Terms] OR "embryo transfer"[Title/Abstract] OR "ET"[Title/Abstract] OR "frozen embryo transfer"[Title/Abstract] OR "FET"[Title/Abstract]) OR  ("Fertility"[MeSH Terms] OR "fertility"[Title/Abstract]) OR  ("Infertility"[MeSH Terms] OR "infertility"[Title/Abstract]) OR  ("Reproductive Techniques, Assisted"[MeSH Terms] OR "assisted reproductive technology"[Title/Abstract] OR "ART"[Title/Abstract]) OR  ("Pregnancy"[MeSH Terms] OR "pregnancy"[Title/Abstract]) OR  ("Abortion, Spontaneous"[MeSH Terms] OR "miscarriage"[Title/Abstract]) OR  ("Embryo Implantation"[MeSH Terms] OR "implantation"[Title/Abstract]) OR  ("Sperm Injections, Intracytoplasmic"[MeSH Terms] OR "intracytoplasmic sperm injection"[Title/Abstract] OR "ICSI"[Title/Abstract]) OR  ("Fertilization in Vitro"[MeSH Terms] OR "in vitro fertilization"[Title/Abstract] OR "IVF"[Title/Abstract])) |  |
| 4 | ((("repeated"[Title/Abstract] OR "multiple"[Title/Abstract] OR "recurrent"[Title/Abstract] OR "frequent"[Title/Abstract] OR "chronic"[Title/Abstract]) OR  (("implantation"[Title/Abstract] OR "engraftment"[Title/Abstract] OR "attachment"[Title/Abstract] OR "nidation"[Title/Abstract]) AND  ("failure"[Title/Abstract] OR "failures"[Title/Abstract] OR "miscarriage"[Title/Abstract] OR "non-success"[Title/Abstract] OR "abort"[Title/Abstract])))  OR  ("RIF"[Title/Abstract] OR "MIF"[Title/Abstract])) |  |
| 5 | #1 AND #2 AND #3 AND #4 | 243 |

- 1. Embase

| **#** | **Searches [<1966-2023]/py** | **Results** |
| --- | --- | --- |
| 1 | ('Chorionic Gonadotropin'/exp OR 'chorionic':ti,ab OR 'gonadotropin':ti,ab OR 'choriogonadotropin':ti,ab OR 'hCG':ti,ab) |  |
| 2 | (('uterus':ti,ab OR 'uterine':ti,ab OR 'endometrium':ti,ab OR 'endometrial':ti,ab OR 'intrauterine':ti,ab OR 'myometrium':ti,ab OR 'cervix':ti,ab OR 'cervical':ti,ab) AND  ('perfusion':ti,ab OR 'infusion':ti,ab OR 'instillation':ti,ab OR 'filling':ti,ab OR 'injection':ti,ab)) |  |
| 3 | (('Embryo Transfer'/exp OR 'embryo transfer':ti,ab OR 'ET':ti,ab OR 'frozen embryo transfer':ti,ab OR 'FET':ti,ab) OR  ('Fertility'/exp OR 'fertility':ti,ab) OR  ('Infertility'/exp OR 'infertility':ti,ab) OR  ('Reproductive Techniques, Assisted'/exp OR 'assisted reproductive technology':ti,ab OR 'ART':ti,ab) OR  ('Pregnancy'/exp OR 'pregnancy':ti,ab) OR  ('Abortion, Spontaneous'/exp OR 'miscarriage':ti,ab) OR  ('Embryo Implantation'/exp OR 'implantation':ti,ab) OR  ('Sperm Injections, Intracytoplasmic'/exp OR 'intracytoplasmic sperm injection':ti,ab OR 'ICSI':ti,ab) OR  ('Fertilization in Vitro'/exp OR 'in vitro fertilization':ti,ab OR 'IVF':ti,ab)) |  |
| 4 | ((('repeated':ti,ab OR 'multiple':ti,ab OR 'recurrent':ti,ab OR 'frequent':ti,ab OR 'chronic':ti,ab) OR  (('implantation':ti,ab OR 'engraftment':ti,ab OR 'attachment':ti,ab OR 'nidation':ti,ab) AND  ('failure':ti,ab OR 'failures':ti,ab OR 'miscarriage':ti,ab OR 'non-success':ti,ab OR 'abort':ti,ab)) OR  ('RIF':ti,ab OR 'MIF':ti,ab)) |  |
| 5 | #1 AND #2 AND #3 AND #4 | 535 |

- 1. Cochrane Library

| **#** | **Searches (with Cochrane Library publication date to Dec 2023, in Trials)** | **Results** |
| --- | --- | --- |
| 1 | ("Chorionic Gonadotropin":ti,ab,kw OR  "chorionic":ti,ab,kw OR  "gonadotropin":ti,ab,kw OR  "choriogonadotropin":ti,ab,kw OR  "hCG":ti,ab,kw OR  "CG":ti,ab,kw) |  |
| 2 | (("uterus":ti,ab,kw OR "uterine":ti,ab,kw OR "endometrium":ti,ab,kw OR "endometrial":ti,ab,kw OR "intrauterine":ti,ab,kw OR "myometrium":ti,ab,kw OR "cervix":ti,ab,kw OR "cervical":ti,ab,kw) AND ("perfusion":ti,ab,kw OR "infusion":ti,ab,kw OR "instillation":ti,ab,kw OR "filling":ti,ab,kw OR "injection":ti,ab,kw)) |  |
| 3 | (("Embryo Transfer":ti,ab,kw OR "embryo transfer":ti,ab,kw OR "ET":ti,ab,kw OR "frozen embryo transfer":ti,ab,kw OR "FET":ti,ab,kw) OR  ("Fertility":ti,ab,kw OR "fertility":ti,ab,kw) OR  ("Infertility":ti,ab,kw OR "infertility":ti,ab,kw) OR  ("Reproductive Techniques, Assisted":ti,ab,kw OR "assisted reproductive technology":ti,ab,kw OR "ART":ti,ab,kw) OR  ("Pregnancy":ti,ab,kw OR "pregnancy":ti,ab,kw) OR  ("Abortion, Spontaneous":ti,ab,kw OR "miscarriage":ti,ab,kw) OR  ("Embryo Implantation":ti,ab,kw OR "implantation":ti,ab,kw) OR  ("Sperm Injections, Intracytoplasmic":ti,ab,kw OR "intracytoplasmic sperm injection":ti,ab,kw OR "ICSI":ti,ab,kw) OR  ("Fertilization in Vitro":ti,ab,kw OR "in vitro fertilization":ti,ab,kw OR "IVF":ti,ab,kw)) |  |
| 4 | ((("repeated":ti,ab,kw OR "multiple":ti,ab,kw OR "recurrent":ti,ab,kw OR "frequent":ti,ab,kw OR "chronic":ti,ab,kw) OR  (("implantation":ti,ab,kw OR "engraftment":ti,ab,kw OR "attachment":ti,ab,kw OR "nidation":ti,ab,kw) AND  ("failure":ti,ab,kw OR "failures":ti,ab,kw OR "miscarriage":ti,ab,kw OR "non-success":ti,ab,kw OR "abort":ti,ab,kw)))  OR  ("RIF":ti,ab,kw OR "MIF":ti,ab,kw)) |  |
| 5 | #1 AND #2 AND #3 AND #4 | 267 |

- 1. Web of Science

| **#** | **Searches (PY=1900-2023)** | **Results** |
| --- | --- | --- |
| 1 | (TS=("Chorionic Gonadotropin" OR chorionic OR gonadotropin OR choriogonadotropin OR hCG OR CG)) |  |
| 2 | (TS=(uterus OR uterine OR endometrium OR endometrial OR intrauterine OR myometrium OR cervix OR cervical) AND TS=(perfusion OR infusion OR instillation OR filling OR injection)) |  |
| 3 | (TS=("Embryo Transfer" OR "embryo transfer" OR "ET" OR "frozen embryo transfer" OR "FET") OR  TS=("fertility") OR  TS=("infertility") OR  TS=("assisted reproductive technology" OR "ART") OR  TS=("pregnancy") OR  TS=("miscarriage") OR  TS=("implantation") OR  TS=("intracytoplasmic sperm injection" OR "ICSI") OR  TS=("in vitro fertilization" OR "IVF")) |  |
| 4 | (TS=("repeated" OR "multiple" OR "recurrent" OR "frequent" OR "chronic") OR  (TS=("implantation" OR "engraftment" OR "attachment" OR "nidation") AND  TS=("failure" OR "failures" OR "miscarriage" OR "non-success" OR "abort")) OR  TS=("RIF" OR "MIF")) |  |
| 5 | #1 AND #2 AND #3 AND #4 | 325 |

- 1. Key Chinese databases

We submitted the Chinese terms 'recurrent implantation failure' and 'Chorionic Gonadotropin' in the search box, and limited the search to Title/Abstract, with the publication date interval extending up to December 31, 2023.

| Database provider | Website | Search results |
| --- | --- | --- |
| CNKI | https://search.cnki.com.cn/ | 55 |
| Wanfang | https://w.wanfangdata.com.cn/ | 11 |
| Weipu | https://qikan.cqvip.com/ | 18 |

**The total number of items from all databases is 1,454. After deduplicating the published articles, only 771 remained. Further screening of the content of these articles resulted in the retention of only 57 articles.**

1. Studies Included After Initial Screening"

| **No.** | **Authors** | **Title** | **Years** | **notes** |
| --- | --- | --- | --- | --- |
| 1 | Mansour, et al. | Intrauterine injection of human chorionic gonadotropin before embryo transfer significantly improves the implantation and pregnancy rates in in vitro fertilization/intracytoplasmic sperm injection: a prospective randomized study | 2011 |  |
| 2 | Cambiaghi, et al. | Intrauterine injection of human chorionic gonadotropin before embryo transfer may improve clinical pregnancy and implantation rates in blastocysts transfers | 2013 |  |
| 3 | Leao, et al. | Intrauterine injection of human chorionic gonadotropin before embryo transfer may improve the pregnancy rates in in vitro fertilization cycles of patients with repeated implantation failures | 2013 |  |
| 4 | Li, et al. | [Intrauterine injection of human chorionic gonadotropin improves the pregnancy rates in in-vitro fertilization-embryo transfer cycles of repeated failures] | 2013 | Chinese |
| 5 | Rebolloso, et al. | Do intrauterine injection of Human Chorionic Gonadotropin (hCG) before embryo transfer increases implantation and pregnancy rates in patients undergoing in vitro fertilization? | 2013 |  |
| 6 | Bhat, et al. | Outcome of intrauterine injection of human chorionic gonadotropin before embryo transfer in patients with previous IVF/ICSI failure: A randomized study | 2014 |  |
| 7 | Hong, et al. | Endometrial infusion of human chorionic gonadotropin at the time of blastocyst embryo transfer does not impact clinical outcomes: a randomized, double-blind, placebo-controlled trial | 2014 |  |
| 8 | Kokkali, et al. | A randomized control pilot study of the use of intrauterine human chorionic gonadotropin injection before embryo transfer in egg recipient cycles | 2014 |  |
| 9 | Santibañez, et al. | Effect of intrauterine injection of human chorionic gonadotropin before embryo transfer on clinical pregnancy rates from in vitro fertilization cycles: a prospective study | 2014 |  |
| 10 | Singh, et al. | Intra-uterine administration of human chorionic gonadotrophin (HCG) before embryo transfer in recurrent implantation failure (RIF) patients improves implantation and pregnancy rates in IVF-ICSI cycles | 2014 |  |
| 11 | Zarei, et al. | Intrauterine administration of recombinant human chorionic gonadotropin before embryo transfer on outcome of in vitro fertilization/ intracytoplasmic sperm injection: A randomized clinical trial | 2014 |  |
| 12 | Aaleyasin, et al. | In vitro Fertilization Outcome following Embryo Transfer with or without Preinstillation of Human Chorionic Gonadotropin into the Uterine Cavity: A Randomized Controlled Trial | 2015 |  |
| 13 | Chung, et al. | Clinical outcomes according to the endometrial HCG infusion time in thawed blastocyst transfer cycles and gene expression profiling of infused endometrium | 2015 |  |
| 14 | Diao, et al. | Endometrial Foxp3+cells: A potential indicator of human chorionic gonadotropin intrauterine perfusion for patients with recurrent implantation failure | 2015 |  |
| 15 | Jung, et al. | Combined treatments of intrauterine perfusion with G-CSF and injection of hCG enhance the endometrial growth, implantation and pregnancy rates in thin endometrium patients | 2015 |  |
| 16 | Li, et al. | Pregnancy outcomes of intrauterine injection of human chorionic gonadotropin before frozen embryo transfer in in vitro fertilization | 2015 |  |
| 17 | Matsumoto, Kokeguchi | Systemic or local human chorionic gonadotropin administration is not beneficial for frozen-thawed embryo transfer in hormone replacement cycles: Two prospective randomized trials | 2015 |  |
| 18 | Wen, et al. | [Effects of intrauterine infusion of human chorionic gonadotropin (hCG) on the clinical outcome before frozen-thawed embryo transfer after repeated implantation failure] | 2015 | Chinese |
| 19 | Wirleitner, et al. | The usefulness of intrauterine hCG administration prior to blastocyst transfer in IVF-patients ≥38 years | 2015 |  |
| 20 | Wirleitner, et al. | Intrauterine administration of human chorionic gonadotropin does not improve pregnancy and life birth rates independently of blastocyst quality: a randomized prospective study | 2015 |  |
| 21 | Dehghani Firouzabadi, et al. | The effect of intrauterine human chorionic gonadotropin injection before embryo transfer on the implantation and pregnancy rate in infertile patients: A randomized clinical trial | 2016 |  |
| 22 | Eskandar, et al. | Does intrauterine injection of human chorionic gonadotropin before embryo transfer improve the pregnancy rate in vitro fertilization/intracytoplasmic sperm injection (IVFICSI) cycles? A prospective randomized controlled trial | 2016 |  |
| 23 | Navali, et al. | Intrauterine administration of hCG immediately after oocyte retrieval and the outcome of ICSI: a randomized controlled trial | 2016 |  |
| 24 | Volovsky, et al. | Intrauterine human chorionic gonadotropin (HCG) infusion prior to embryo transfer (ET) may be detrimental to pregnancy rate | 2016 |  |
| 25 | Dutta | A randomized study showing the outcome of intrauterine injection of human chorionic gonadotropin before embryo transfer in patients with previous IVF/ICSI failure | 2017 |  |
| 26 | Huang, et al. | A study of intrauterine infusion of human chorionic gonadotropin (hCG) before frozen-thawed embryo transfer after two or more implantation failures | 2017 |  |
| 27 | Kanter, et al. | Intrauterine injection of HCG to enhance embryo implantation | 2017 |  |
| 28 | Mostajeran, et al. | Effect of intrauterine injection of human chorionic gonadotropin before embryo transfer on pregnancy rate: A prospective randomized study | 2017 |  |
| 29 | Aly, et al. | The effect of timing intrauterine human chorionic gonadotropin injection before embryonic transfer on intracytoplasmic sperm injection outcomes: A prospective randomized study | 2018 |  |
| 30 | Dutta | Outcome of intrauterine injection of HCG before et in patients with previous IVF/ICSI failure | 2018 |  |
| 31 | Hafezi, et al. | The effect of intrauterine human chorionic gonadotropin flushing on live birth rate after vitrified-warmed embryo transfer in programmed cycles: a randomized clinical trial | 2018 |  |
| 32 | Huang, et al. | Effects of intrauterine perfusion of human chorionic gonadotropin in women with different implantation failure numbers | 2018 |  |
| 33 | Huang, et al. | [Different intrauterine perfusion in patients with frozen-thawed embryo transfer after implantation failure] | 2018 | Chinese |
| 34 | Ma, et al. | [Effect of Intrauterine Perfusion of hCG before Embryo Transfer on Pregnancy Outcome in Patients with FET Repeated Implantation Failure] | 2018 | Chinese |
| 35 | Volovsky, et al. | Should intrauterine human chorionic gonadotropin infusions ever be used prior to embryo transfer? | 2018 |  |
| 36 | Laokirkkiat, et al. | Increased implantation rate after intrauterine infusion of a small volume of human chorionic gonadotropin at the time of embryo transfer: a randomized, double-blind controlled study | 2019 |  |
| 37 | Liu, et al. | Intrauterine administration of human chorionic gonadotropin improves the live birth rates of patients with repeated implantation failure in frozen-thawed blastocyst transfer cycles by increasing the percentage of peripheral regulatory T cells | 2019 |  |
| 38 | Wang, et al. | [Intrauterine injection of human chorionic gonadotropin improves pregnancy outcome in patients with repeated implantation failure in frozen-thawed embryo transfer] | 2019 | Chinese |
| 39 | Xiaomin, et al. | The molecular mechanism of human chorionic gonadotropin (hCG) increasing the endometrial Tregs in women with RIF | 2019 |  |
| 40 | Huang, et al. | [Effect of hCG Intrauterine Administration for different age groups of Repeated Implantation Failure] | 2020 | Chinese |
| 41 | Tong, et al. | [Effect of intrauterine HCG infusion on pregnancy outcome of repeated implantation failure in frozen thawed embryo transfer cycles] | 2020 | Chinese |
| 42 | Zhao, et al. | [Effects of intrauterine infusion of HCG on pregnancy outcome in patients with repeated implantation failure in freeze-thaw embryo transfer cycles] | 2020 | Chinese |
| 43 | Abdallah, et al. | Intrauterine injection of HCG before embryo transfer: a parallel, double-blind randomized trial | 2021 |  |
| 44 | Asbagh, et al. | The Effect of Intrauterine Infusion of Human Gonadotropin (HCG) on the Outcome of Embryo Transfer Cycles in Infertile Women: A Randomized Clinical Trial | 2021 |  |
| 45 | Hosseinisadat, et al. | Effects of human chorionic gonadotropin intrauterine injection on oocyte retrieval day on assisted reproductive techniques outcomes: An RCT | 2021 |  |
| 46 | Ji, et al. | [Effect of intrauterine perfusion of HCG on pregnancy outcome in patients with repeated implantation failures] | 2021 | Chinese |
| 47 | Li, et al. | [Effect of intrauterine infusion of human chorionic gonadotropin on pregnancy outcome after freeze-thaw embryo transfer in patients with repeated transplant failure] | 2021 | Chinese |
| 48 | Xiong, et al. | [Effect of HCG intrauterine perfusion before frozen-thawed blastocyst transplantation on pregnancy outcome in patients with recurrent implantation failure of IVF-ET] | 2021 | Chinese |
| 49 | Atef, et al. | Value of Intrauterine Injection of HCG (Human Chorionic Gonadotropin) before Fresh Embryo Transfer on Clinical Pregnancy Rate in Women with Previous Failed One or Two ICSI (Intra Cytoplasmic Sperm Injection) Trials | 2022 |  |
| 50 | Bakry, et al. | Granulocyte colony stimulating factor versus human chorionic gonadotropin for recurrent implantation failure in intra cytoplasmic sperm injection: a randomized clinical trial | 2022 |  |
| 51 | Cheng, et al. | [Effect of intrauterine perfusion in patients with frozen-thawed embryo transfer after recurrent implantation failures] | 2022 | Chinese |
| 52 | Li, et al. | [Effects of the intrauterine perfusion of recombinant hCG before a frozen-thawed embryo transfer in women with different implantation failure times] | 2022 | Chinese |
| 53 | Liu, et al. | [Effect of intrauterine perfusion with rHCG during FET after RIF on pregnancy outcome] | 2022 | Chinese |
| 54 | Mei, et al. | Clinical outcome of intrauterine administration of peripheral mononuclear cells or human chorionic gonadotropin in unexplained implantation failure | 2022 |  |
| 55 | Mustapha, et al. | Effect of intrauterine administration of human chorionic gonadotropin one day before fresh blastocyst transfer on clinical outcomes: a quasi-experimental study | 2022 |  |
| 56 | Torky, et al. | Effect of Intra Uterine Granulocyte Colony Stimulating Factor vs Human Chorionic Gonadotropin at Ovum Pick Up Day on Pregnancy Rate in IVF/ICSI Cases With Recurrent Implantation Failure | 2022 |  |
| 57 | Xu, et al. | [Effect of Different Intrauterine Perfusion Schemes on Pregnancy Outcome of Frozen Thawed Embryo Transfer in Patients with Repeated Implantation Failure] | 2023 | Chinese |

1. Detailed Exclusion of Non-Compliant Studies

| **No.** | **Authors** | **Title** | **Years** | **notes** |
| --- | --- | --- | --- | --- |
| 1 | Bakry M S, et al. | Granulocyte colony stimulating factor versus human chorionic gonadotropin for recurrent implantation failure in intra cytoplasmic sperm injection: a randomized clinical trial | 2022 | No reliable control |
| 2 | Leao, et al. | Intrauterine injection of human chorionic gonadotropin before embryo transfer may improve the pregnancy rates in in vitro fertilization cycles of patients with repeated implantation failures | 2013 | Incomplete data |
| 3 | Li TT, et al. | [Intrauterine injection of human chorionic gonadotropin improves the pregnancy rates in in-vitro fertilization-embryo transfer cycles of repeated failures] | 2013 | Incomplete data |
| 4 | Tong, et al. | [Effect of intrauterine HCG infusion on pregnancy outcome of repeated implantation failure in frozen thawed embryo transfer cycles] | 2020 | Incomplete data |
| 5 | Wirleitner, et al. | The usefulness of intrauterine hCG administration prior to blastocyst transfer in IVF-patients ≥38 years | 2015 | Non-RIF studies |
| 6 | Matsumoto, Kokeguchi | Systemic or local human chorionic gonadotropin administration is not beneficial for frozen-thawed embryo transfer in hormone replacement cycles: Two prospective randomized trials | 2015 | Non-RIF studies |
| 7 | Volovsky, et al. | Should intrauterine human chorionic gonadotropin infusions ever be used prior to embryo transfer? | 2018 | Non-RIF studies |
| 8 | Kokkali, et al. | A randomised control pilot study of the use of intrauterine human chorionic gonadotropin injection before embryo transfer in egg recipient cycles | 2014 | Non-RIF studies |
| 9 | Li, et al. | Pregnancy outcomes of intrauterine injection of human chorionic gonadotropin before frozen embryo transfer in in vitro fertilization | 2015 | Non-RIF studies |
| 10 | Mansour, et al. | Intrauterine injection of human chorionic gonadotropin before embryo transfer significantly improves the implantation and pregnancy rates in in vitro fertilization/intracytoplasmic sperm injection: a prospective randomized study | 2011 | Non-RIF studies |
| 11 | Cambiaghi, et al. | Intrauterine injection of human chorionic gonadotropin before embryo transfer may improve clinical pregnancy and implantation rates in blastocysts transfers | 2013 | Non-RIF studies |
| 12 | Kanter, et al. | Intrauterine injection of HCG to enhance embryo implantation | 2017 | Non-RIF studies |
| 13 | Abdallah, et al. | Intrauterine injection of HCG before embryo transfer: a parallel, double-blind randomized trial | 2021 | Non-RIF studies |
| 14 | Volovsky, et al. | Intrauterine human chorionic gonadotropin (HCG) infusion prior to embryo transfer (ET) may be detrimental to pregnancy rate | 2016 | Non-RIF studies |
| 15 | Zarei, et al. | Intrauterine administration of recombinant human chorionic gonadotropin before embryo transfer on outcome of in vitro fertilization/ intracytoplasmic sperm injection: A randomized clinical trial | 2014 | Non-RIF studies |
| 16 | Wirleitner, et al. | Intrauterine administration of human chorionic gonadotropin does not improve pregnancy and life birth rates independently of blastocyst quality: a randomised prospective study | 2015 | Non-RIF studies |
| 17 | Navali, et al. | Intrauterine administration of hCG immediately after oocyte retrieval and the outcome of ICSI: a randomized controlled trial | 2016 | Non-RIF studies |
| 18 | Laokirkkiat, et al. | Increased implantation rate after intrauterine infusion of a small volume of human chorionic gonadotropin at the time of embryo transfer: a randomized, double-blind controlled study | 2019 | Non-RIF studies |
| 19 | Aaleyasin, et al. | In vitro Fertilization Outcome following Embryo Transfer with or without Preinstillation of Human Chorionic Gonadotropin into the Uterine Cavity: A Randomized Controlled Trial | 2015 | Non-RIF studies |
| 20 | Hong, et al. | Endometrial infusion of human chorionic gonadotropin at the time of blastocyst embryo transfer does not impact clinical outcomes: a randomized, double-blind, placebo-controlled trial | 2014 | Non-RIF studies |
| 21 | Hosseinisadat, et al. | Effects of human chorionic gonadotropin intrauterine injection on oocyte retrieval day on assisted reproductive techniques outcomes: An RCT | 2021 | Non-RIF studies |
| 22 | Aly, et al. | The effect of timing intrauterine human chorionic gonadotropin injection before embryonic transfer on intracytoplasmic sperm injection outcomes: A prospective randomized study | 2018 | Non-RIF studies |
| 23 | Mostajeran, et al. | Effect of intrauterine injection of human chorionic gonadotropin before embryo transfer on pregnancy rate: A prospective randomized study | 2017 | Non-RIF studies |
| 24 | Santibañez, et al. | Effect of intrauterine injection of human chorionic gonadotropin before embryo transfer on clinical pregnancy rates from in vitro fertilisation cycles: a prospective study | 2014 | Non-RIF studies |
| 25 | Asbagh, et al. | The Effect of Intrauterine Infusion of Human Gonadotropin (HCG) on the Outcome of Embryo Transfer Cycles in Infertile Women: A Randomized Clinical Trial | 2021 | Non-RIF studies |
| 26 | Dehghani Firouzabadi, et al. | The effect of intrauterine human chorionic gonadotropin injection before embryo transfer on the implantation and pregnancy rate in infertile patients: A randomized clinical trial | 2016 | Non-RIF studies |
| 27 | Hafezi, et al. | The effect of intrauterine human chorionic gonadotropin flushing on live birth rate after vitrified-warmed embryo transfer in programmed cycles: a randomized clinical trial | 2018 | Non-RIF studies |
| 28 | Mustapha, et al. | Effect of intrauterine administration of human chorionic gonadotropin one day before fresh blastocyst transfer on clinical outcomes: a quasi-experimental study | 2022 | Non-RIF studies |
| 29 | Eskandar, et al. | Does intrauterine injection of human chorionic gonadotropin before embryo transfer improve the pregnancy rate in vitro fertilization/intracytoplasmic sperm injection (IVFICSI) cycles? A prospective randomized controlled trial | 2016 | Non-RIF studies |
| 30 | Rebolloso, et al. | Do intrauterine injection of Human Chorionic Gonadotropin (hcg) before embryo transfer increases implantation and pregnancy rates in patients undergoing in vitro fertilization? | 2013 | Non-RIF studies |
| 31 | Jung, et al. | Combined treatments of intrauterine perfusion with G-CSF and injection of hCG enhance the endometrial growth, implantation and pregnancy rates in thin endometrium patients | 2015 | Non-RIF studies |
| 32 | Chung, et al. | Clinical outcomes according to the endometrial HCG infusion time in thawed blastocyst transfercycles and gene expression profiling of infused endometrium | 2015 | Non-RIF studies |
| 33 | Xiaomin, et al. | The molecular mechanism of human chorionic qgonadotropin (hCG) increasing the endometrial Tregs in women with RIF | 2019 | Non-clinical studies |
| 34 | Diao, et al. | Endometrial Foxp3+cells: A potential indicator of human chorionic gonadotropin intrauterine perfusion for patients with recurrent implantation failure | 2015 | Non-clinical studies |
| 35 | Atef, et al. | Value of Intrauterine Injection of HCG (Human Chorionic Gonadotropin) before Fresh Embryo Transfer on Clinical Pregnancy Rate in Women with Previous Failed One or Two ICSI (Intra Cytoplasmic Sperm Injection) Trials | 2022 | Definition of RIF: <3 |
| 36 | Huang, et al. | A study of intrauterine infusion of human chorionic gonadotropin (hCG) before frozen-thawed embryo transfer after two or more implantation failures | 2017 | Definition of RIF: <3 |
| 37 | Ma, et al. | [Effect of Intrauterine Perfusion of hCG before Embryo Transfer on Pregnancy Outcome in Patients with FET Repeated Implantation Failure] | 2018 | Definition of RIF: <3 |
| 38 | Huang, et al. | [Effect of hCG Intrauterine Administration for different age groups of Repeated Implantation Failure] | 2020 | Definition of RIF: <3 |
| 39 | Huang, et al. | [Different itrauterine perfusion in patients with frozen-thawed embryo transfer after implantation failure] | 2018 | Definition of RIF: <3 |
| 40 | Mei, et al. | Clinical outcome of intrauterine administration of peripheral mononuclear cells or human chorionic gonadotropin in unexplained implantation failure | 2022 | Definition of RIF: <3 |
| 41 | Dutta | A randomized study showing the outcome of intrauterine injection of human chorionic gonadotropin before embryo transfer in patients with previous IVF/ICSI failure | 2017 | Lack of RIF definition |
| 42 | Bhat, et al. | Outcome of intrauterine injection of human chorionic gonadotropin before embryo transfer in patients with previous IVF/ICSI failure: A randomized study | 2014 | Lack of RIF definition |
| 43 | Dutta | Outcome of intrauterine injection of HCG before et in patients with previous IVF/ICSI failure | 2018 | Lack of RIF definition |
| 44 | Singh, et al. | Intra-uterine administration of human chorionic gonadotrophin (HCG) before embryo transfer in recurrent implantation failure (RIF) patients improves implantation and pregnancy rates in IVF-ICSI cycles | 2014 | Lack of RIF definition |

1. Included Studies for Meta-analysis

| Publication Year | Author | Title | Journal | URL |
| --- | --- | --- | --- | --- |
| 2022 | Cheng LL, et al. | [Effect of intrauterine perfusion in patients with frozen-thawed embryo transfer after recurrent implantation failures] | *Journal of Reproductive Medicine* | <https://qikan.cqvip.com/Qikan/Article/Detail?id=7108347792> |
| 2018 | Huang PX, et al. | Effects of intrauterine perfusion of human chorionic gonadotropin in women with different implantation failure numbers | *American Journal of Reproductive Immunology* | <https://pubmed.ncbi.nlm.nih.gov/29288552/> |
| 2021 | Ji XY, et al. | [Effect of intrauterine perfusion of HCG on pregnancy outcome in patients with repeated implantation failures] | *Journal of Southeast University: Medical Science Edition* | <https://qikan.cqvip.com/Qikan/Article/Detail?id=7104264272> |
| 2022 | Li J, et al. | [Effects of the intrauterine perfusion of recombinant hCG before a frozen-thawed embryo transfer in women with different implantation failure times] | *Journal of Henan University: Medical Science* | <https://qikan.cqvip.com/Qikan/Article/Detail?id=00002GCMK7387JP0MLDO4JP1M9R> |
| 2021 | Li R, et al. | [Effect of intrauterine infusion of human chorionic gonadotropin on pregnancy outcome after freeze-thaw embryo transfer in patients with repeated transplant failure] | *Journal of Guangxi Medical University* | <https://qikan.cqvip.com/Qikan/Article/Detail?id=7105026697> |
| 2022 | Liu N, et al. | [Effect of intrauterine perfusion with rHCG during FET after RIF on pregnancy outcome] | *Chinese Journal of Human Sexuality* | <https://qikan.cqvip.com/Qikan/Article/Detail?id=7107109701> |
| 2019 | Liu XM, et al. | Intrauterine administration of human chorionic gonadotropin improves the live birth rates of patients with repeated implantation failure in frozen-thawed blastocyst transfer cycles by increasing the percentage of peripheral regulatory T cells | *Archives of Gynecology and Obstetrics* | <https://pubmed.ncbi.nlm.nih.gov/30659362/> |
| 2022 | Torky H, et al. | Effect of Intra Uterine Granulocyte Colony Stimulating Factor vs Human Chorionic Gonadotropin at Ovum Pick Up Day on Pregnancy Rate in IVF/ICSI Cases With Recurrent Implantation Failure | *JBRA assisted reproduction* | <https://pubmed.ncbi.nlm.nih.gov/34786904/> |
| 2019 | Wang M, et al. | [Intrauterine injection of human chorionic gonadotropin improves pregnancy outcome in patients with repeated implantation failure in frozen-thawed embryo transfer] | *Journal of Central South University: Medical Science (Zhong Nan Da Xue Xue Bao Yi Xue Ban)* | <https://pubmed.ncbi.nlm.nih.gov/31919319/> |
| 2015 | Wen Y, et al. | [Effects of intrauterine infusion of human chorionic gonadotropin (hCG) on the clinical outcome before frozen-thawed embryo transfer after repeated implantation failure] | Reproduction and Contraception | <https://qikan.cqvip.com/Qikan/Article/Detail?id=663989802> |
| 2021 | Xiong YL, et al. | [Effect of HCG intrauterine perfusion before frozen-thawed blastocyst transplantation on pregnancy outcome in patients with recurrent implantation failure of IVF-ET] | Journal of Reproductive Medicine | <https://qikan.cqvip.com/Qikan/Article/Detail?id=7104598635> |
| 2023 | Xu DJ, et al. | [Effect of Different Intrauterine Perfusion Schemes on Pregnancy Outcome of Frozen Thawed Embryo Transfer in Patients with Repeated Implantation Failure] | Medical Innovation of China | <https://qikan.cqvip.com/Qikan/Article/Detail?id=7110054621> |
| 2020 | Zhao SF, et al. | [Effects of intrauterine infusion of HCG on pregnancy outcome in patients with repeated implantion failure in freeze-thaw embryo transfer cycles] | Journal of China Prescription Drug | <https://qikan.cqvip.com/Qikan/Article/Detail?id=7103258526> |

1. NOS Assesment Criteria

To evaluate the quality of studies on hCG intrauterine perfusion in patients with recurrent implantation failure (RIF) after three or more failed embryo transfers, we use the Newcastle-Ottawa Scale (NOS).

**Representative:**

We first consider the representativeness of the study population. For cohort studies, the ideal study population should include all eligible RIF patients or a specific group of RIF patients from a particular hospital or clinic. For case-control studies, the study subjects should be newly diagnosed or first-time treated RIF patients or consecutively enrolled RIF patients. Studies with good representativeness can more accurately reflect the overall situation.

**Selection:**

In terms of selection, we require that the control group in case-control studies be randomly sampled from the same community or hospital non-RIF patients, with the method of control group selection clearly described. The selection of the control group must ensure good comparability to accurately assess the effect of hCG intrauterine perfusion.

**Ascertainment of exposure:**

For the ascertainment of exposure, studies need to confirm the implementation of hCG intrauterine perfusion through valid tools such as medical records or structured interviews. This ensures the accuracy and reliability of the data, whereas self-reports are relatively less ideal. Studies that do not clearly describe the method of exposure confirmation will receive lower scores in this category.

**Demonstration:**

Regarding outcome demonstration, for cohort studies, subjects should not have achieved clinical pregnancy at the beginning of the study. This ensures that all study subjects start from the same baseline state, thereby increasing the validity of the study results. Studies that do not exclude subjects with existing clinical pregnancies at the start will receive lower scores in this category.

**Comparability:**

In terms of comparability, we require that studies control for important confounding factors such as age, BMI, and baseline endocrine function. Additionally, they should control for other confounding factors such as the patient's previous treatment history and lifestyle. These control measures can reduce the impact of confounding variables on the study results, thereby increasing the credibility of the results.

**Outcome:**

For the outcome category, cohort studies need to confirm clinical pregnancy results through independent review or record linkage, while case-control studies should conduct blind assessment. Results confirmed by these methods are more reliable, whereas self-reported results are relatively weaker. Studies that do not clearly describe the method of outcome confirmation will receive lower scores in this category.

**Follow-up:**

The adequacy of follow-up is also a crucial evaluation standard. Cohort studies need to ensure a follow-up rate of ≥70% to maintain data integrity and representativeness. If the follow-up rate is insufficient and there is a possibility of bias in the comparative analysis, it affects the credibility of the study results. Studies with a low follow-up rate will receive lower scores in this category.

**Adequacy of follow-up:**

Regarding the adequacy of follow-up, cohort studies should have less than 20% of study subjects lost to follow-up. A high proportion of lost to follow-up may lead to bias and affect the credibility of the results. Studies with a high proportion of lost to follow-up will receive lower scores in this category.

Finally, we score each study based on these standards to ensure fair evaluation of the quality of each study included in the meta-analysis. High-quality studies will receive the highest scores, indicating excellent design and data quality; moderate-quality studies will have relatively high scores but not full marks; low-quality studies will have fewer stars, indicating poorer design and data quality. Through this systematic and standardized evaluation method, we ensure the reliability and validity of the meta-analysis results.

1. Egger Test for Publication Bias

| **Study** | **t_value** | **df_value** | **p_value** |
| --- | --- | --- | --- |
| Implantation | 0.114903 | 10 | 0.910796 |
| Clinical pregnancy | -0.06782 | 11 | 0.947145 |
| Miscarriage | -0.5916 | 9 | 0.568678 |
